# Comprehensive analysis of pyroptosis‐related gene signatures for glioblastoma immune microenvironment and target therapy

**DOI:** 10.1111/cpr.13376

**Published:** 2023-01-21

**Authors:** Zeyu Wang, Ziyu Dai, Hao Zhang, Nan Zhang, Xisong Liang, Luo Peng, Jian Zhang, Zaoqu Liu, Yun Peng, Quan Cheng, Zhixiong Liu

**Affiliations:** ^1^ Department of Neurosurgery, Xiangya Hospital Central South University Changsha China; ^2^ National Clinical Research Center for Geriatric Disorders Changsha China; ^3^ MRC Centre for Regenerative Medicine, Institute for Regeneration and Repair University of Edinburgh Edinburgh UK; ^4^ One‐Third Lab, College of Bioinformatics Science and Technology Harbin Medical University Harbin China; ^5^ Department of Oncology, Zhujiang Hospital Southern Medical University Guangzhou China; ^6^ Department of Interventional Radiology The First Affiliated Hospital of Zhengzhou University Zhengzhou China; ^7^ Department of Geriatrics, Xiangya Hospital Central South University Changsha China; ^8^ Teaching and Research Section of Clinical Nursing Xiangya Hospital of Central South University Changsha China

## Abstract

Glioblastoma (GBM) is a malignant brain tumour, but its subtypes (mesenchymal, classical, and proneural) show different prognoses. Pyroptosis is a programmed cell death relating to tumour progression, but its association with GBM is poorly understood. In this work, we collected 73 GBM samples (the Xiangya GBM cohort) and reported that pyroptosis involves tumour‐microglia interaction and tumour response to interferon‐gamma. GBM samples were grouped into different subtypes, cluster 1 and cluster 2, based on pyroptosis‐related genes. Cluster 1 samples manifested a worse prognosis and had a more complicated immune landscape than cluster 2 samples. Single‐cell RNA‐seq data analysis supported that cluster 1 samples respond to interferon‐gamma more actively. Moreover, the machine learning algorithm screened several potential compounds, including nutlin‐3, for cluster 1 samples as a novel treatment. In vitro experiments supported that cluster 1 cell line, T98G, is more sensitive to nutlin‐3 than cluster 2 cell line, LN229. Nutlin‐3 can trigger oxidative stress by increasing DHCR24 expression. Moreover, pyroptosis‐resistant genes were upregulated in LN229, which may participate against nutlin‐3. Therefore, we hypothesis that GBM may be able to upregulate pyroptosis resistant related genes to against nutlin‐3‐triggered cell death. In summary, we conclude that pyroptosis highly associates with GBM progression, tumour immune landscape, and tumour response to nutlin‐3.

## Graphical abstract

We collected 73 glioblastoma samples, as well as single‐cell and bulk RNA‐seq data from public databases, combined with a machine‐learning approach to explore the impact of focal death on glioma prognosis, and validated it through in‐vitro experiments. Nutlin‐3 can trigger oxidative stress by increasing DHCR24 expression. Moreover, pyroptosis‐resistant genes were upregulated in LN229, which may participate against nutlin‐3. In summary, we conclude that pyroptosis highly associates with glioblastoma progression, tumour immune landscape and tumour response to nutlin‐3.

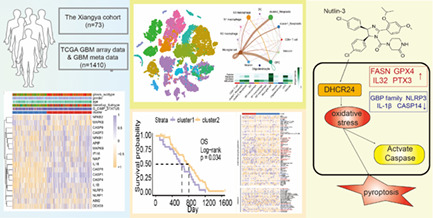

## 1 INTRODUCTION

Glioblastoma (GBM) is the most malignant tumour deriving from the central nervous system with poor survival outcomes. Classification based on genomic characteristics of GBM successfully grouped tumours into different groups, like the Verhaak subtype.^1^, ^2^ This classification proposed three subtypes, including mesenchymal, classical, and proneural of GBM and viewed mesenchymal GBM as the most aggressive subtype. Moreover, research reported that mesenchymal GBM tended to be invasive from immune system surveillance and proposed this difference may result from abnormal transcription factor activation.^3^ Therefore, the excavation of the genomic characteristic of GBM assists in understanding the difference between GBM subtypes and discovering novel therapeutic targets.

Pyroptosis is an inflammatory associated with programmed cell death involving microorganism invasion, central nervous system diseases, and atherosclerosis. Pyroptosis is triggered by the formation of the inflammasome, which can induce cell membrane pores and result in cell death. Caspase‐1‐dependent pathway, the canonical pathway, can be activated by the inflammasome, and caspase‐1 can cause cell membrane pores through cleaving gasdermin D (GSDMD). Besides, other noncanonical pathways like caspase‐4/GSDMD pathway, caspase‐5/GSDMD, and caspase‐3/gasdermin E (GSDME) can also trigger pyroptosis. Pyroptotic cells release massive inflammatory‐associated factors to recruit or activate immunocytes. Together, pyroptosis plays a critical role in modulating cell death and the activation of the immune system.

Pyroptosis has been widely reported in its role in tumour progression, including colorectal cancer, ovarian cancer, gastric cancer, and lung cancer.^4^, ^5^, ^6^ For instance, the expression of GSDMD and GSDME is altered in gastric cancer than in normal tissue indicating pyroptosis may involve tumour progression.^7^, ^8^, ^9^ MiR‐214 can suppress glioma proliferation and migration by targeting caspase‐1.^10^ NLRP3 inflammasome and AIM2 inflammasome were reported that participate in hepatocellular carcinoma progression by affecting pyroptosis.^11^, ^12^ Increased TP53 expression suppressed lung cancer progression through inducing pyroptosis.^13^ On the other hand, pyroptosis is also connected to tumour resistance to chemotherapy, like malignant mesothelioma,^14^ gastric cancer,^8^ and lung cancer,^15^ which might attribute to pyroptotic cells releasing inflammatory factors (like IL‐1β). Pyroptosis is tightly associated with tumour progression, but its association with GBM is poorly understood.

Interferon‐gamma, type II interferon, is a product of lymphocytes^16^ and have been widely reported in affecting tumour immune microenvironment^17^, ^18^ and tumour response to immunotherapy, including haematologic malignancies,^19^ GBM,^20^, ^21^ and lung adenocarcinoma.^22^ Moreover, several studies reported that interferon‐gamma can trigger pyroptosis.^23^, ^24^, ^25^ For instance, pyroptosis activation can be modulated by interferon‐gamma‐secreted macrophage.^25^ Interferon‐gamma upregulates GSDMB to promote pyroptosis.^23^ Therefore, exploring the connection of interferon‐gamma with pyroptosis in GBM may assist in understanding how to control GBM progression.

In this work, samples from our database, the Xiangya GBM cohort, were grouped based on the critical regulators of pyroptosis with consensus clustering analysis. Two clusters were grouped, cluster 1 and cluster 2, and cluster 1 samples manifested a worse prognosis and a more complicated immune landscape. Those results were cross‐verified in the TCGA GBM array dataset and GBM meta dataset contains 1410 samples from different datasets. Moreover, single‐cell RNA‐seq data were also analysed to view the potential interaction between tumour cells and immunocytes. We noticed that cluster 2 samples communicated with microglia through multiple pathways and had higher score on tumor response to interferon‐gamma. To step further, we screened potential drugs for GBM with different pyroptosis gene expression maps, and results were verified in the GBM cell line. Compound, nutlin‐3, may trigger GBM pyroptosis by causing oxidative stress, and cell lines in different clusters respond to it differentially by modulating pyroptosis associated genes' expression. Taken together, this work identified the role of pyroptosis in GBM and proposed the potential mechanism of how pyroptosis affects tumour immune landscape.

## 2 METHODS AND MATERIALS

### 2.1 Genomic data

We collected GBM samples to construct the Xiangya GBM cohort (https://ngdc.cncb.ac.cn/, China National Center for Bioinformation, ID: HRA001618) and used it as a training cohort^26^ (Glioma tissues were collected, and written informed consent was obtained from all patients. The included glioma tissues were approved by the Ethics Committee of Xiangya Hospital, Central South University).

TCGA GBM array data (https://xenabrowser.net/) and GBM meta dataset (1410 samples), which combined data from TCGA (RNA‐seq data of GBM), Chinese Glioma Genome Atlas (CGGA, http://www.cgga.org.cn/) and Gene Expression Omnibus (GEO, https://www.ncbi.nlm.nih.gov/geo/) were used to validate results from Xiangya GBM cohort. For the GBM meta dataset, the batch effect was processed before merging different datasets. The subtype of GBM (mesenchymal, classical, neural, and proneural) was provided from the TCGA database, and the gliovis_subtype of GBM (mesenchymal, classical, and proneural) was predicted by Gliovis (http://gliovis.bioinfo.cnio.es/).^27^

Single‐cell RNA‐seq analysis analysed 8953 cells from 33 GBM samples (SCP50 and SCP393), and data were obtained from the Single Cell Portal platform (http://singlecell.broadinstitute.org). Quality control of each data was set as: mitochondrial genes on account of total genes <15%; haemoglobin genes on account of total genes <5%; the number of total genes for each cell >500. Then, data from different sources were integrated by R package ‘Seurat’. Tumour cells were identified by R package ‘infercnv’. Cell types were determined by R package ‘scCATCH’. The central nervous system tumour cell line expression profile was downloaded from cancer cell line encyclopaedia (CCLE).

### 2.2 Pyroptosis‐associated model construction

Consensus clustering analysis was performed on GBM based on pyroptosis critical regulators, and two groups were obtained, cluster 1 and cluster 2 (R package ‘ConsensusClusterPlus’).^28^ Parameters were set as follow: distance = ‘pearson’, maxK = 10, reps = 1000, pItem = 0.8, pFeature = 1, clusterAlg = ‘pam’. A similar strategy was also applied to TCGA GBM array data and GBM meta dataset.

### 2.3 Machine learning

The cluster model was constructed in the central nervous system tumour cell line with multiple machine‐learning algorithms. Support Vector Machines, Shrunken Centroids Classifier, and Stuttgart Neural Network Simulator were performed with R package ‘e1071’, ‘pamr’, and ‘RSNNS’, respectively.

### 2.4 Tumour immunogenicity and cell–cell communication

Gene ontology (GO) and Kyoto encyclopaedia of genes and genomes (KEGG) analyses were performed to view the differential potential bio function pathways between cluster 1 and 2. GO, and KEGG analysis was conducted by employing gene set variation analysis (GSVA) and gene set enrichment analysis (GSEA) with the R package ‘GSVA’^29^ and ‘clusterProfiler’,^30^ respectively.

Two types of immunogram were introduced to evaluate tumour immunogenicity, as previously reported.^31^, ^32^ Pathways including T cell immunity, absence of inhibitory molecules, absence of checkpoint expression, absence of inhibitory cells, recognition of tumour cells, trafficking and infiltration, priming and activation, innate immunity, interferon‐gamma response, proliferation, and glycolysis were evaluated by using ssGSEA with R package ‘GSVA’.

The gene set of immune escape‐related genes was obtained from the previous study.^33^ As for single‐cell RNA‐seq analysis, genes that were not detected in over 80% of all samples were excluded.

The tumour immune landscape was depicted by the ESTIMATE algorithm, CIBERSORT algorithm, and xCELL algorithm. The ESTIMATE algorithm and xCELL algorithm were performed with R package ‘ESTIMATE’^34^ and ‘xCell’,^35^ respectively. The CIBEERSORT algorithm was performed as guided in https://cibersortx.stanford.edu/.^36^, ^37^

R package ‘CellChat’ was introduced to predict cell–cell communication based on ligand‐receptor pairs between tumour cells and immunocytes.^38^ Significant differential ligand‐receptors pairs between cluster 1 and cluster 2 were selected.

### 2.5 Drug prediction

Information on tumour cell line sensitivity to potential drugs was downloaded from cancer therapeutics response portal version 1 and version 2 (CTRP v1 and CTRP v2)^39^, ^40^, ^41^ and profiling relative inhibition simultaneously in mixtures (PRISM).^42^ The lower AUC of the cell line indicates a higher sensitivity to potential drugs. The expression of the tumour cell line was downloaded from CCLE. The prediction was conducted with the R package ‘pRRophetic’.^43^ Differential sensitivity drugs were identified with R package ‘limma’. The protocol was performed as previously reported.^44^

### 2.6 Cell culture

GBM cell lines (T98G and LN229) were purchased from BeNa Culture Collection (https://www.bncc.org.cn/) along with STR qualification. Cells cultured in a cell incubator at 37°C and 5% CO_2_. The culture medium consisted of high glucose DMEM and 10% FBS, and the culture medium was changed every 3 days. Cells were digested with trypsin and passaged when cells were grown at 80%–90% confluency.

### 2.7 The calculation of IC50

Cells were seeded in 96 wells with a density of 2000 cells per well. Nutlin‐3 was purchased from Selleck (https://www.selleck.cn/). Cells were treated with different concentration of nulin‐3 (90 μM, 80 μM, 70 μM, 60 μM, 50 μM, 40 μM, 30 μM, 20 μM, 10 μM) for 48 h. The CCK8 assay was performed to determine the cell survival ratio and calculate IC50. A total of 10% CCK8 reagent was added to each well, and the optical density value at 450 nm was measured.

### 2.8 Cell growth

The proliferation ability of T98G and LN229 was also further verified by the CCK8 assay and the colony‐forming assay as previously described.^45^

### 2.9 Quantitative polymerase chain reaction

Cells were treated with nutlin‐3 for 48 h. Then, DNA extraction and PCR were performed as previously described.^45^ Primers were listed below:

GAPDH: forward primer: ACAGCCTCAAGATCATCAGC; reverse primer: GGTCATGAGTCCTTCCACGAT.

DHCR24: forward primer: TGAAGACAAACCGAGAGGGC; reverse primer: CAGCCAAAGAGGTAGCGGAA.

### 2.10 RNA‐seq sample preparation

All samples were run in triplicate. Cells were collected after treatment with nutilin‐3 for 48 h. RNA integrity was determined by the RNA Nano 6000 Assay Kit of the Bioanalyzer 2100 system (Agilent Technologies, CA, USA).

Briefly, 1 ug RNA per sample was prepared for further analysis. Poly‐T oligo‐attached magnetic beads were used to obtain purified mRNA from total RNA. Using divalent cations under elevated temperature, Fragmentation was carried out in First Strand Synthesis Reaction Buffer (5X). First and second strand cDNA was synthesized subsequently using random hexamer primer and M‐MuLV Reverse Transcriptase (RNase H) and using DNA Polymerase I and RNase H, respectively. The remaining overhangs were cleaved into blunt ends by exonuclease/polymerase activities. After adenylation of 3′ ends of DNA fragments, Adaptor with a hairpin loop structure was ligated to prepare for hybridization. Purified library fragments by the AMPure XP system (Beckman Coulter, Beverly, USA) were applied to select cDNA fragments at the length of preferentially 370–420 bp. PCR was performed subsequently with Universal PCR primers, Phusion High‐Fidelity DNA polymerase, and Index (X) Primer. Finally, PCR products were purified (also by the AMPure XP system), and library quality was evaluated by the Agilent Bioanalyzer 2100 system.

The clustering of the index‐coded samples was performed on a cBot cluster generation system using TruSeq PE Cluster Kit v3‐cBot‐HS (Illumia) as per the manufacturer's instructions. After cluster generation, the library preparations were sequenced on an Illumina Novaseq platform which eventually generated 150 bp paired‐end reads. All data were transformed into log2(TPM + 1) for subsequent analysis.

### 2.11 Statistical analysis

Normality Test was performed on those datasets first. For normally distributed data, student *t*‐test and one‐way ANOVA tests were conducted to compare the difference between two or multiple groups. As for non‐normally distributed data, the comparison between two groups or within multiple groups was examined with the Wilcox test or the Kruskal–Wallis test, respectively. Cell subtype composition between cluster 1 and cluster 2 was examined with the Chi‐square test. The Kaplan–Meier analysis and log‐rank test were performed for overall survival analysis. The CCK8 assay was examined with a two‐way ANOVA test. IC50 was calculated by GraphPad Prism (version 8.0.2). All bioinformatics information was carried out with R (version 3.6.1).

## 3 RESULTS

### 3.1 Cluster 1 GBM manifested worse clinical outcome than cluster 2 GBM

**FIGURE 1:**
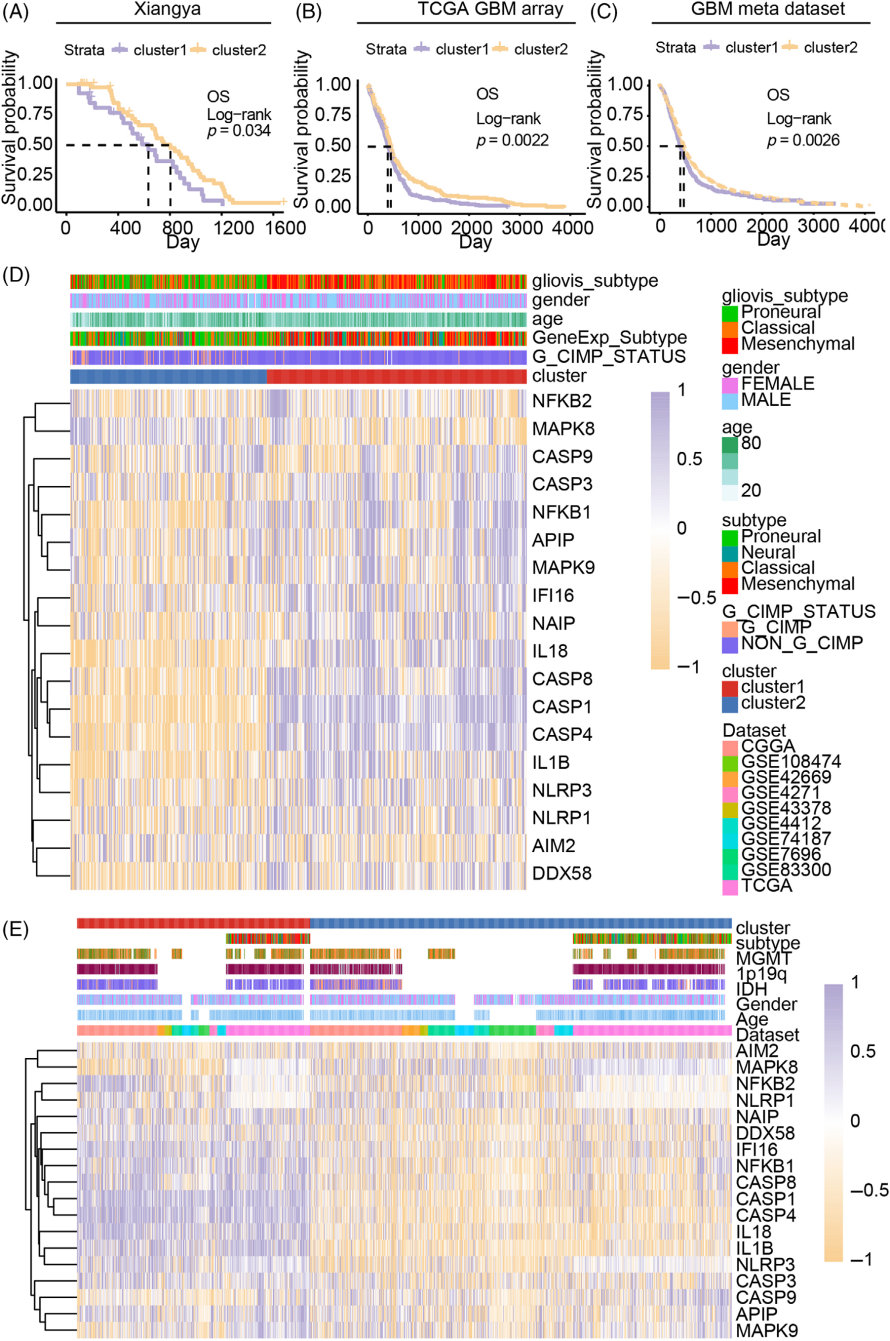
Association of the cluster mode with glioblastoma (GBM) prognosis. (A) Overall survival analysis of the cluster model in the Xiangya GBM cohort. (B) Overall survival analysis of the cluster model in the TCGA GBM array cohort. (C) Overall survival analysis of the cluster model in the GBM meta dataset. Heatmap shows the association of the cluster model with GBM clinical features and pyroptosis‐related genes expression in TCGA GBM array data (D) and GBM meta dataset (E). NS, no significant; *p < 0.05; **p < 0.01; ***p < 0.001.

Pyroptosis critical regulators were identified from previous studies and filtered out if they are not detected in different datasets. Two clusters, cluster 1 and cluster 2, were classified (Figure S1A‐C) based on 18 pyroptosis‐related genes (including CASP1, CASP3, CASP4, CASP8, CASP9. AIM2, APIP, IL1B, IL18, IFI16, DDX58, NFKB1, NFKB2, NLRP1, NLRP3, NAIP, MAPK8, and MAPK9).^5^, ^46^ Kaplan–Meier overall survival analysis suggested that samples from cluster 1 manifested worse clinical outcomes than cluster 2 in Xiangya GBM cohort (*p* = 0.0034, Figure 1A), TCGA GBM array dataset (*p* = 0.0022, Figure 1B), and GBM meta dataset (*p* = 0.0026, Figure 1C). Disease‐specific survival analysis (*p* = 0.002, Figure S3A) and progression‐free interval survival analysis (*p* = 0.0035, Figure S3B) were further performed, and cluster 1 was also verified as malignant subtype.

In Xiangya GBM cohort, the expression of APIP, CASP1, CASP4, CASP8, DDX58, IL1B, IL18, NFKB1, and NLRP3 in cluster 1 samples was higher than that in cluster 2 samples. Meanwhile, the expression of AIM2, CASP9 and MAPK8 increased in cluster 2 samples (Figure S2). The expression of pyroptosis activation‐associated genes like caspase‐1, caspase‐3, caspase‐8, IL‐1β, IL‐18, and NLRP3 were increased while AIM2 was decreased in cluster 1 than in cluster 2 (Figure 1D). The classification was further validated in GBM meta dataset, and similar gene expression profile alternation was also identified (Figure 1E). In the meantime, aggressive subtypes like mesenchymal and classical mainly were enriched in cluster 1 (Figure S3C,D). Therefore, the expression of pyroptosis activation‐associated regulators was increased in cluster 1, indicating pyroptosis may modulate GBM progression.

### 3.2 More malignant GBM cells in cluster 1 GBM cells than that in cluster 2

**FIGURE 2:**
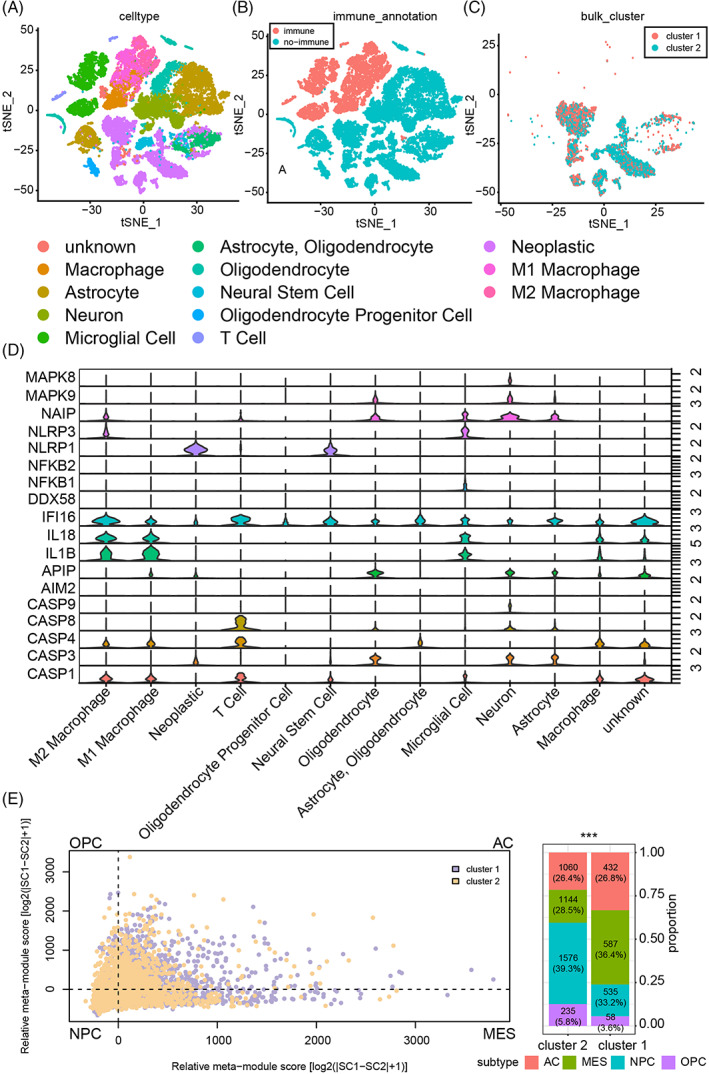
The cluster model with single‐cell RNA‐seq analysis. (A) and (B) Cell types in single‐cell RNA‐seq analysis. (C) The cluster model in neoplastics in single‐cell RNA‐seq analysis. (D) The expression profile of pyroptosis‐associated genes in single‐cell RNA‐seq analysis. (E) The association of the cluster model with neoplastic subtypes. NS, no significant; *p < 0.05; **p < 0.01; ***p < 0.001.

Single‐cell RNA‐seq analysis was further introduced, and the composition of cell subtype in GBM samples was also mapped (Figure 2A,B). The cluster model was built based on neoplastic with a support vector machine algorithm (Figure 2C). The expression profile of pyroptosis critical regulators was also depicted, and most of them showed higher expression in neoplastic and immunocytes like macrophage and microglia than other stromal cells (Figure 2D).

We predicted the GBM cells subtype as previously described.^47^ As illustrated, more MES‐like (mesenchymal‐like) cells and AC‐like (astrocyte‐like) cells were labelled as cluster 1 cells, and more NPC‐like (neural‐progenitor‐like) and OPC‐like (oligodendrocyte‐progenitor‐like) cells were classified as cluster 2 cells (Figure 2E). Therefore, cluster 1 GBM cells are more malignant than cluster 2 GBM cells since GBM samples with a higher proportion of MES‐like and AC‐like cells manifest poor prognosis.

### 3.3 Cluster 1 GBM showed a remarkable difference in the activation of immunocytes‐associated pathways

**FIGURE 3:**
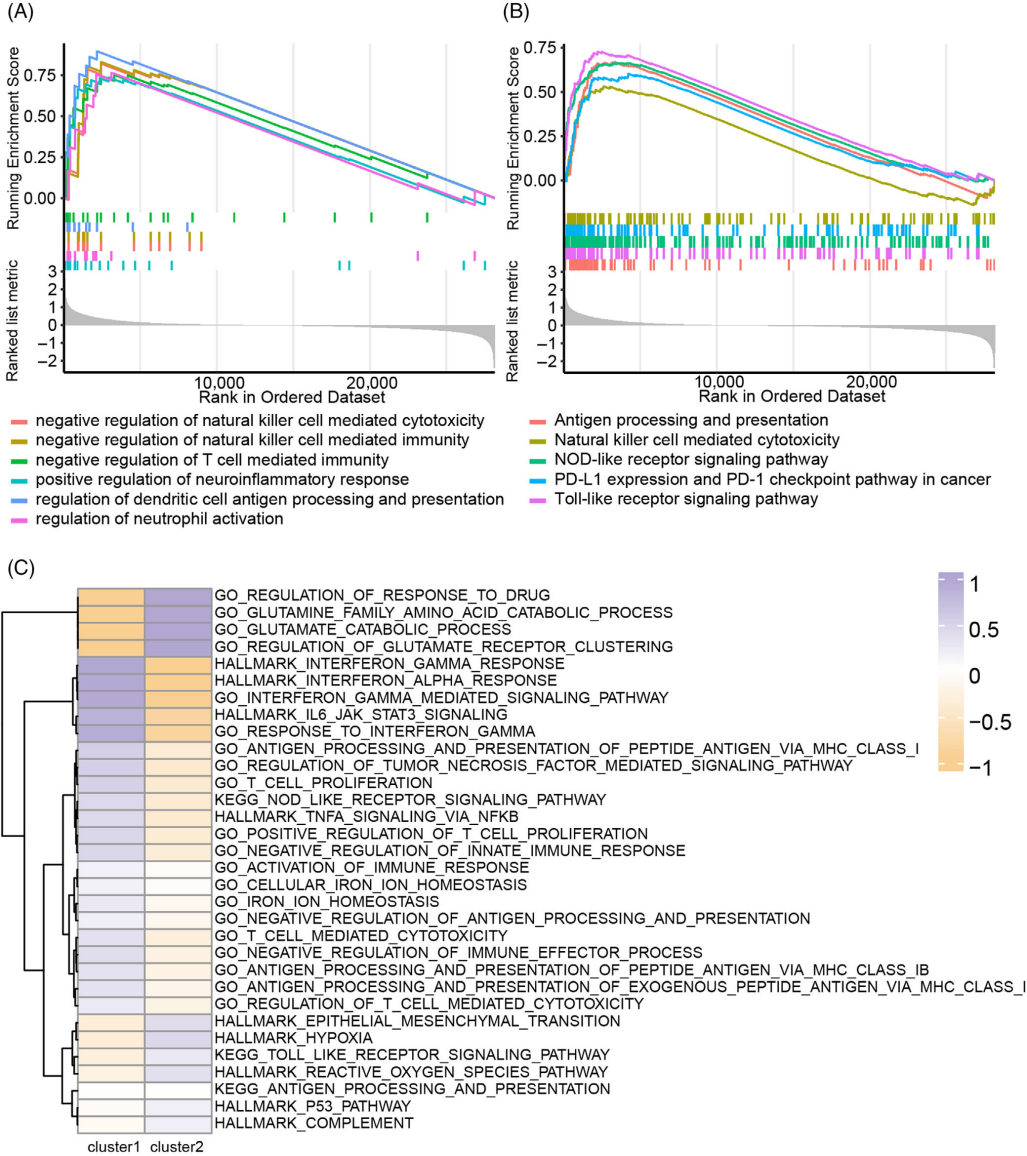
Biofunction enrichment prediction. (A) Gene ontology (GO) enrichment analysis based on gene set enrichment analysis (GSEA) analysis in the Xiangya GBM cohort. (B) Kyoto encyclopaedia of genes and genomes (KEGG) enrichment analysis based on GSEA analysis in the Xiangya GBM cohort. (C) GO, KEGG, and HALLAMRK enrichment analysis based on GSEA analysis in single‐cell RNA‐seq analysis.

GO and KEGG analysis based on GSEA were performed to examine the difference between cluster 1 and cluster 2. In Xiangya GBM cohort, GO analysis suggested that the regulation of immunocytes, like NK cells and T cells, and antigen presentation‐associated pathways were activated in cluster 1 samples (Figure 3A). KEGG analysis showed that the regulation of natural killer cells and T cells, PD‐L1 and PD‐1 associated pathways, and the NOD‐like and TOLL‐like pathways were differentially activated in cluster 1 samples (Figure 3B). GO and KEGG analysis based on GSEA were also performed in TCGA GBM array dataset (Figure S4A) and GBM meta dataset (Figure S4B). Pathways like NK cells and T cells related pathway, antigen representation‐related pathways, tumour response to interferon, Toll‐like signalling pathway, PD‐L1, and PD‐1 related pathway were enriched in cluster 1.

GO and KEGG analysis based on GSEA in neoplastic from single‐cell RNA‐seq analysis were performed (Figure 3C). Tumour response to interferon‐gamma and interferon‐alpha, IL6 pathway, antigen representation related pathway, and T cell‐related pathway were enriched in cluster 1 samples while glutamine‐related pathway, tumour response to drug and P53 pathway were activated in cluster 2 samples. Therefore, cluster 1 samples are highly associated with immunocytes related pathways indicating the role of pyroptosis activation in tumour immune landscape.

### 3.4 Tumour immunogenicity and immune checkpoints' expression difference in cluster 1 and 2 GBM cells

**FIGURE 4:**
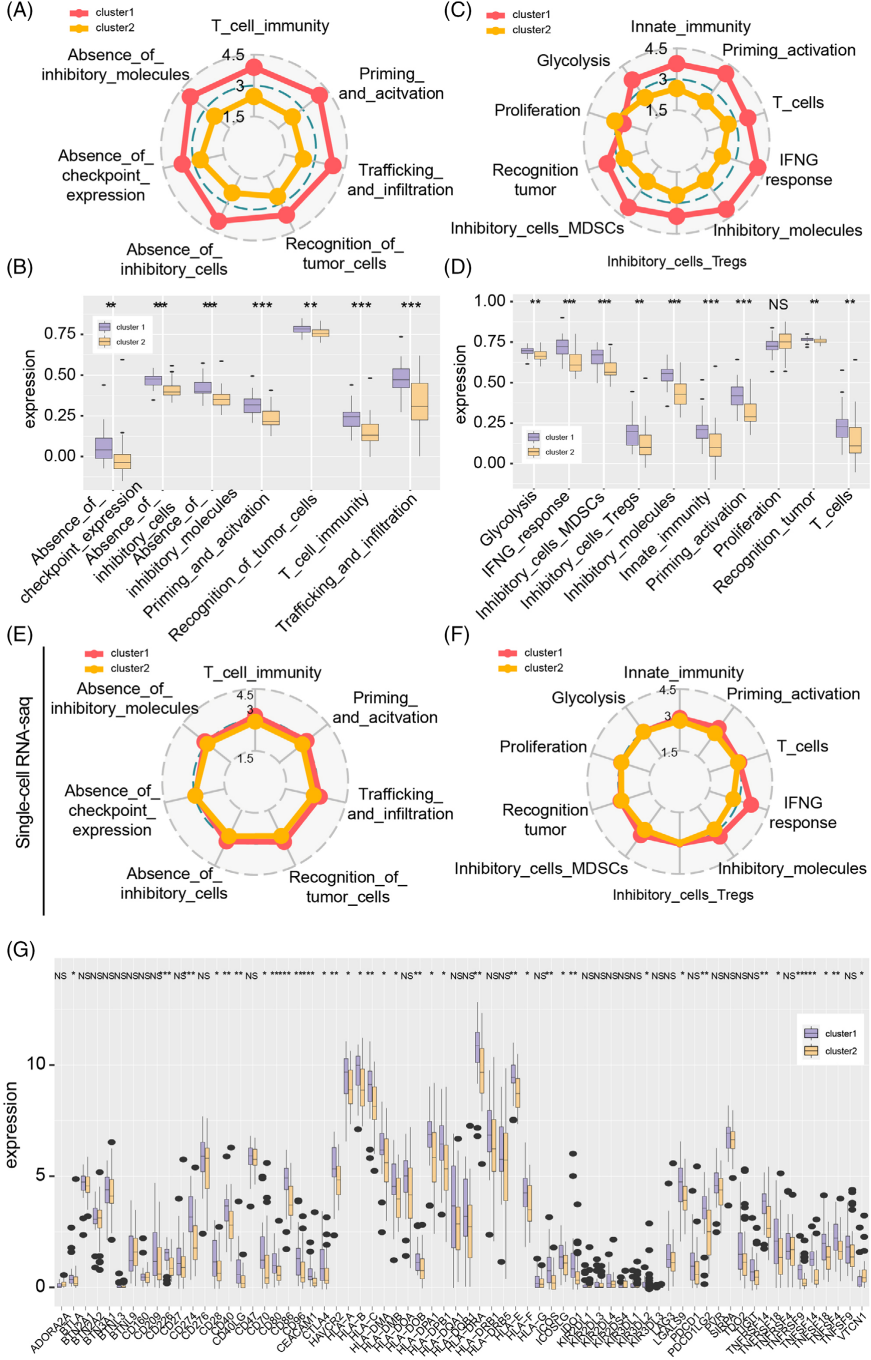
Immunogenicity and immune checkpoint genes. (A) and (B) The construction of immunogram (version 2017) in the Xiangya GBM cohort. (C) and (D) The construction of immunogram (version 2020) in the Xiangya GBM cohort. (E) and (F) The construction of immunogram in the single‐cell RNA‐seq data. (G) Immune checkpoint genes' expression in the Xiangya GBM cohort. NS, no significant; *p < 0.05; **p < 0.01; ***p < 0.001.

Immunogenicity was evaluated by mapping immunogram as previously performed.^31^, ^32^, ^48^ In Xiangya GBM cohort, cluster 1 had a higher score on immunogenicity than cluster 2, including T cells immunity, glycolysis, recognition tumour, interferon‐gamma response, tumour cells recognition, infiltration of inhibitory myeloid‐derived suppressor cells and regulatory T cells, the absence of inhibitory immunocytes, and absence of immune checkpoint expression (Figure 4A–D). Similar tumour immunogenicity was also observed in the GBM meta dataset (Figure S5A). However, no significant difference in the ‘absence of checkpoint expression’ between cluster 1 and cluster 2 was observed in the TCGA GBM array dataset (Figure S5B). Moreover, the difference in ‘absence of checkpoint expression’, ‘glycolysis’, ‘inhibitory cells (Tregs)’, ‘Proliferation’, ‘recognition tumour’ and ‘T cells’ were not found between cluster 1 and cluster 2 GBM cells in single‐cell RNA‐seq GBM analysis (Figures 4E,F, and S5C).

The expression profile of immune escape‐related genes was mapped. Higher expression of genes like BTLA, CD226, CD274, CD28, CD40, CD40LG, CD70, CTLA4, and PDCD1LG2 was noticed in cluster 2 than in cluster 1 in Xiangya GBM cohort (Figure 4G). In single‐cell RNA‐seq GBM data, the higher expression profiles of CD276, CD47, and MHC like HLA‐A, HLA‐B, and HLA‐C were also noticed in cluster 1 neoplastic (Figure S6A). A similar expression profile can be noticed in the TCGA GBM array and GBM meta dataset (Figure S6B,C). Therefore, the difference of immunogenicity and immune escape‐related genes between cluster 1 and cluster 2 samples indicates pyroptosis may affect tumour immunosuppressive microenvironment.

### 3.5 Higher infiltration ratio of macrophages in cluster 1 samples than in cluster 2 samples

**FIGURE 5:**
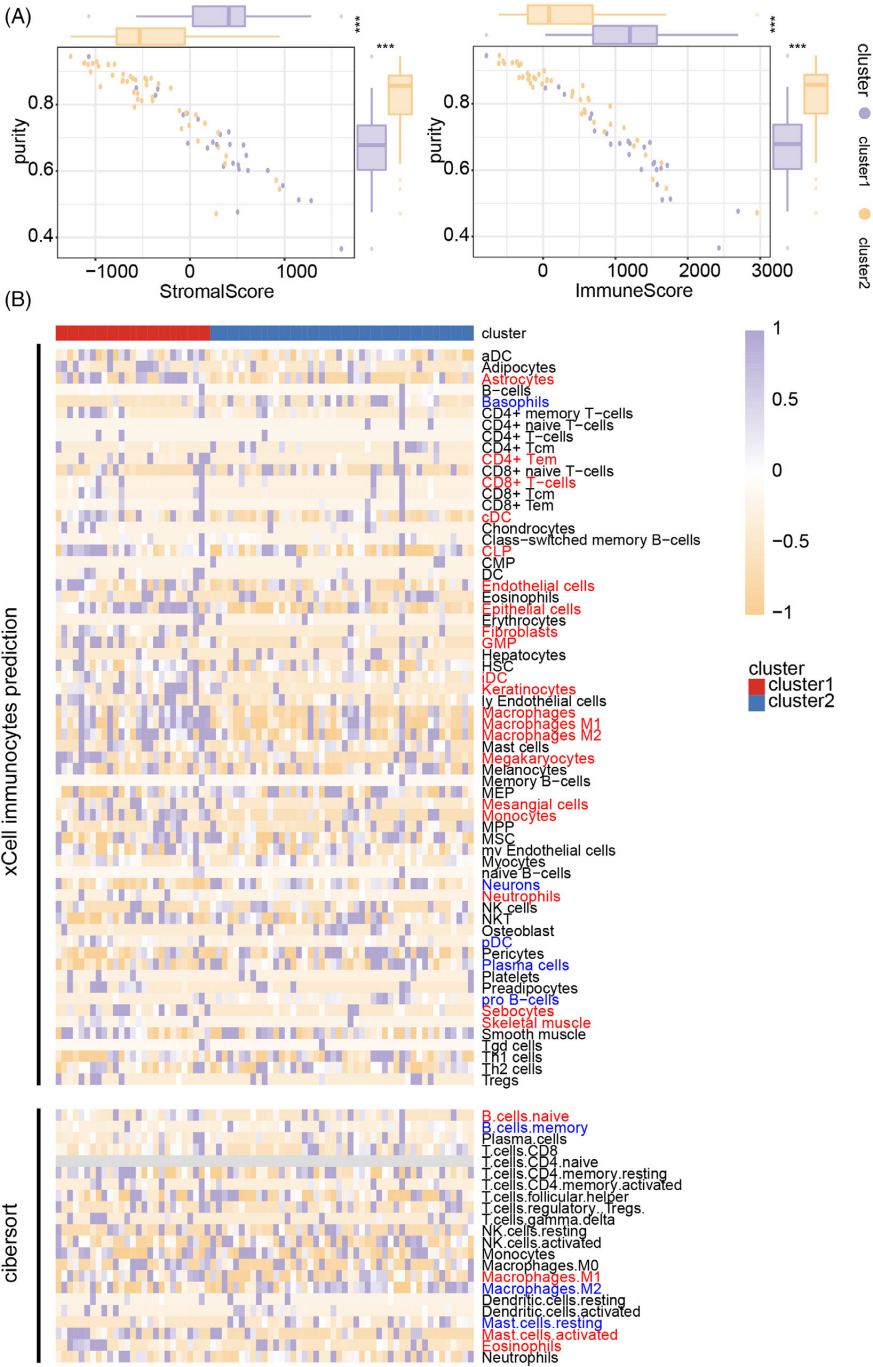
Immune landscape difference in the cluster model. (A) Correlation of tumour purity with stromal score or immune score was calculated in the Xiangya GBM cohort by performing the ESTIMATE algorithm. (B) Immunocytes infiltration in Xiangya GBM cohort by using the CIBERSORT algorithm and xCell analysis.

Next, we explored the immune landscape difference between clusters 1 and 2. More immunocytes and stromal cell infiltrated in samples from cluster 1 than cluster 2 according to the ESTIMATE algorithm in Xiangya GBM cohort (Figure 5A), TCGA GBM array dataset (Figure S7A), and GBM meta dataset (Figure S8A).

Then, the infiltration map of immunocytes and stromal cells was further mapped. Immunocytes preferentially infiltrated in cluster 1 GBM samples were marked as red while cells infiltrated in cluster 2 GBM samples were marked as blue in Figure 5. Immunocytes like M1 macrophage, CD4+ memory T cells, neutrophils, and dendritic cells in cluster 1 samples were predicted, while a lower infiltration ratio of dendritic cells and Basophils was found in Xiangya GBM cohort (Figure 5B). Nevertheless, M0 and M1 macrophages were enriched in cluster 1 samples according to the xCell algorithm, but the CIBERSORT algorithm suggested differently. In the TCGA GBM array dataset, macrophage and M1 macrophage were enriched in cluster 1 samples. More T cells like CD4 naïve T cells and CD8 naïve T cells were found in cluster 2 samples (Figures S7B and S8B). Taken together, the immunocytes infiltration in cluster 1 samples was more complicated than in cluster 2.

### 3.6 Cluster 2 GBM cells interacted with microglia through multiple pathways

**FIGURE 6:**
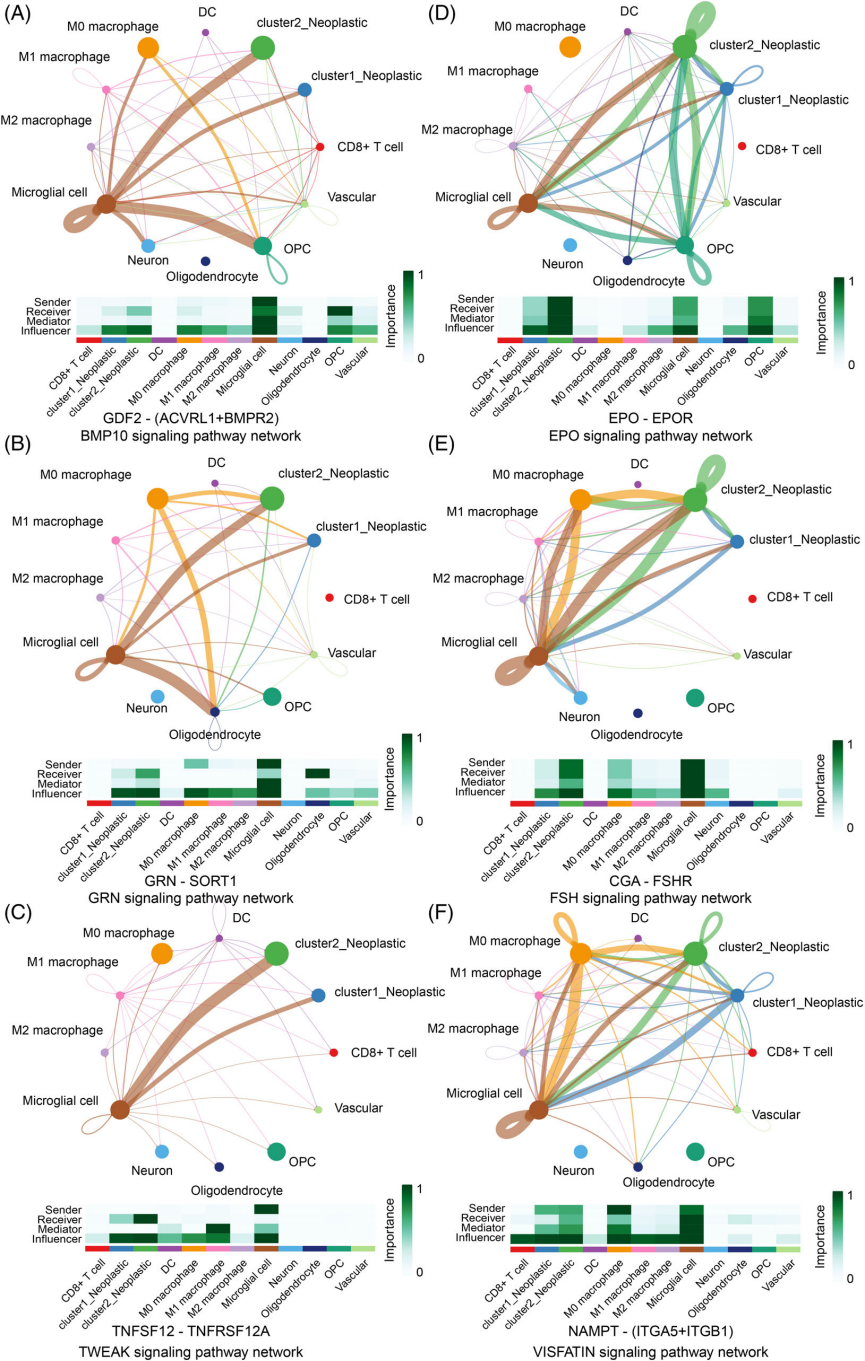
Cell–cell communication differences in the cluster model. (A) GDF2‐(ACVRL+BMPR2) in the BMP10 signalling pathway. (B) GRN‐SORT1 in the GRN signalling pathway. (C) TNFSF12‐TNFSF12A in the TWEAK signalling pathway. (D) EPO‐EPOR in the EPO signalling pathway. (E) CGA‐FSHR in the FSH signalling pathway. (F) NAMPT‐(ITGA5 + ITGB1) in the VISFATIN signalling pathway.

We investigated cell–cell communication between neoplastic and immunocytes since the tumour immune landscape was different. Generally, cluster 2 GBM cells interacted with microglia through multiple pathways. Novel ligand‐receptor pairs like GDF2‐(ACVRL1 + BMPR2) (Figure 6A), GRN‐SORT1 (Figure 6B), TNFSF12‐TNFRSF12A (Figure 6C), EPO‐EPOR (Figure 6D), CGA‐FSHR (Figure 6E), and NAMPT‐(ITGA5 + ITGB1) (Figure 6F) was first identified between GBM cells and immunocytes. Besides, cluster 2 neoplastic can also communicate with M0 macrophage through GRN‐SORT1, CGA‐FSHR and NAMPT‐(ITGA5 + ITGB1). As illustrated, neoplastic mostly acted as the influencer of microglia (a detailed introduction about their roles can be found in reference [38, 49]).

Several ligand‐receptor pairs were reported previously, like HBEGF‐EGFR (Figure S9A), SPP1‐CD44 (Figure S9B), OSM‐(LIFR+IL6ST) (Figure S9C), FASL–FAS (Figure S9D), IGF1‐(ITGA6 + ITGB4) (Figure S9E), IL6‐(IL6R + IL6ST) (Figure S9F). All those pairs suggested that cluster 2 neoplastic communicated with microglia through multiple pathways. Moreover, cluster 2 neoplastic also acted as a sender, mediator, and influencer to modulate microglia through NGF‐NGFR (Figure S10A), KITL‐KIT (Figure S10B), and KLK3‐NGFR (Figure S10C). Besides, cluster 2 neoplastic communicated with CD8+ T cells through GZMA‐F2R (Figure S10D).

### 3.7 Cluster 1 GBM showed higher sensitivity to nutlin‐3 than cluster 2 GBM

**FIGURE 7:**
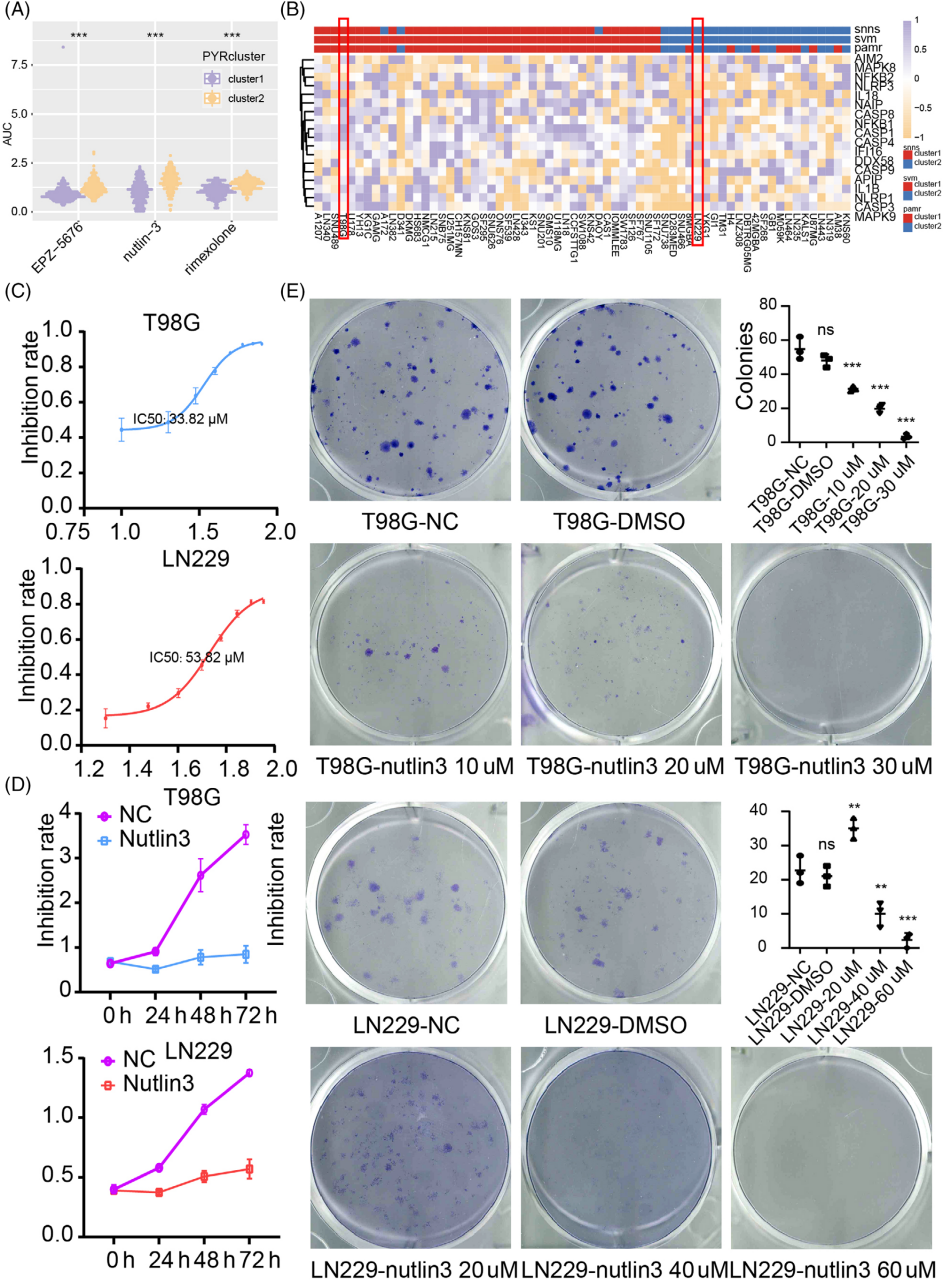
Potential targeted drugs prediction. (A) Drug prediction based on the CTRP and PRISM dataset. (B) Central nervous system tumour cell lines classification is based on machine learning. (C) IC50 of nutlin‐3 of T98G and LN229. (D) The proliferation ability of T98G and LN229 by using CCK8 assay. (E) The clonogenicity of U251MG, T98G, and LN229 by using a colony‐forming assay. NS, no significant; **p < 0.01; ***p < 0.001.

We predicted cluster 1 and cluster 2 GBM cells' sensitivity to different compounds, and three compounds (EPZ‐5676, nutlin‐3, and rimexolone) were identified (Figure 7A). We verified the Central nervous system tumour cell lines' sensitive to nutlin‐3 by calculating IC50. Central nervous system tumour cell lines were classified into cluster 1 or cluster 2 by performing machine learning, Stuttgart Neural Network Simulator, Support Vector Machine, and Shrunken Centroids Classifier. GBM cell line T98G was classified as cluster 1 cells, while GBM cell line LN229 was characterized as cluster 2 cells (Figure 7B). The IC50 of nutlin‐3 for T98G and LN229 was 34.93 μM (34.93 ± 1.92) and 57.35 μM (57.35 ± 3.17), implying the cluster model can be applied in screening potential sensitive drugs for GBM (Figure 7C).

Nutlin‐3 targets the interaction of MDM2‐p53 by inhibiting MDM2 activity and inducing p53 expression, and increased p53 expression can promote pyroptosis.^13^, ^50^ Therefore, the IC50 of nutlin‐3 further proved that the activation of pyroptosis in cluster 1 samples might be more feasible. Then, we confirmed that nutlin‐3 could inhibit GBM cell progression by using the CCK8 assay and the colony‐forming assay (Figure 7D,E).

### 3.8 Nutlin‐3 upregulated DHCR24 to cause oxidative stress and induce cell death

**FIGURE 8:**
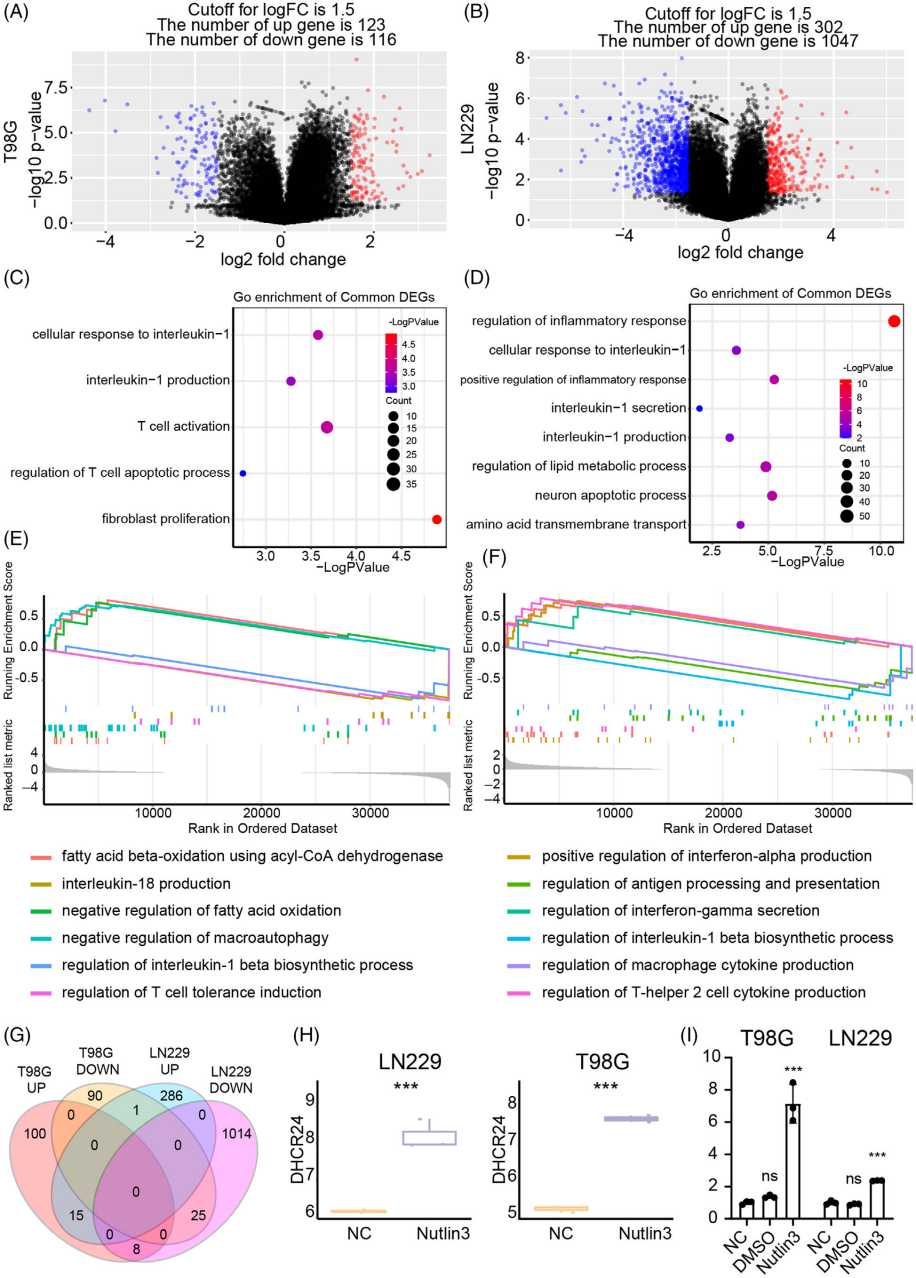
RNA‐seq data analysis based on nutlin‐3‐treated cell line. (A) Differential expression genes before and after treating T98G with nutlin‐3. (B) Differential expression genes before and after treating LN229 with nutlin‐3. (C) Gene ontology (GO) enrichment analysis based on nutlin‐3‐treated T98G. (D) GO enrichment analysis based on nutlin‐3 treated LN229. (E) GO enrichment analysis based on gene set enrichment analysis (GSEA) analysis in nutlin‐3‐treated T98G. (F) GO enrichment analysis based on GSEA analysis in nutlin‐3‐treated LN229. (G) Interaction of differential expression genes in T98G and LN229. (H) The expression of DHCR24 in T98G and LN229 after treating with nutlin‐3 from RNA‐seq. (I) DHCR expression in T98G and LN229 after treating with nutlin‐3 from qPCR. NS, no significant; ***p < 0.001.

Next, we treated T98G and LN229 with nutlin‐3 for 48 hours and prepared for RNA‐seq. Differentially expressed genes were identified by comparing nutlin‐3 group and NC group in T98G (Figure 8A) and LN229 (Figure 8B). GO enrichment analysis based on DEGs suggested that pathways like a cellular response to IL‐1, IL‐1 production, T cell activation, and regulation of T cell apoptotic process were activated in nutlin‐3 treated T98G (Figure 8C), while regulation of inflammatory response, regulation of lipid metabolic process and amino acid transmembrane transport were enriched in nutlin‐3 treated LN229 (Figure 8D). GO enrichment analysis based on the GSEA algorithm showed that pathways like fatty acid beta‐oxidation using acyl‐CoA dehydrogenase, IL‐18 production, negative regulation of fatty acid oxidation, and regulation of IL‐1β biosynthetic process were found in nutlin‐3 treated T98G (Figure 8E). In the meantime, pathways like positive regulation of interferon‐α production, regulation of antigen processing and presentation, regulation of interferon‐gamma secretion, and regulation of macrophage cytokine production were activated in nutlin‐3 treated LN229 (Figure 8F).

Then, we explored critical changes on pyroptosis associated regulators' expression in nutlin‐3 treated cell lines. We found that the expression of DHCR24 increase in nutlin‐3 treated T98G and LN229by intersecting DEGs from T98G and LN229 (Figure 8G,H, Table S1). Meanwhile, results from qPCR also supported that the expression of DHCR24 is upregulated after treating T98G and LN229 with nutlin‐3 (Figure 8I). DHCR24 can trigger oxidative stress and modulate caspase‐3, caspase‐9 activation by interfering cholesterol biosynthesis.^51^, ^52^ Oxidative stress can also trigger cell death through pyroptosis. Therefore, nutlin‐3 may induce the upregulation of DHCR24 to cause oxidative stress and trigger pyroptosis.

Meanwhile, pyroptosis‐resistant genes like FASN,^53^ GPX4^54^ were also upregulated in nutlin‐3 treated LN229 (Figure S10E), and the expression of pyroptosis sensitivity genes like IL32^55^ and PTX3^56^ was increased in nutlin‐3‐treated T98G (Figure S10F). Moreover, the expression of pyroptosis sensitivity genes like the GBP family,^57^ NLRP3, IL‐1β, and CASP14^58^ was decreased in nutlin‐3 treated LN229 (Figure S10G). Together, LN229 may upregulate pyroptosis‐resistant genes and downregulate pyroptosis‐sensitivity genes against that process.^59^

## 4 DISCUSSION

Pyroptosis activation involved in tumour progression, including oesophageal carcinoma,^60^ myeloid leukaemia,^61^ and GBM,^62^ and its regulators were viewed as a potential therapeutic targeted marker.^63^, ^64^ In our work, we defined two types of GBM cells, cluster 1 and cluster 2, and cluster 1 samples were characterized as malignant GBM subtypes (mesenchymal and classical GBM, according to a previous study reported^1^, ^2^). Moreover, the higher consistency of mesenchymal GBM cells in GBM samples based on single‐cell RNA‐seq analysis also supported that theory. In the meantime, the expression of pyroptosis initiation regulators like NLRP3, AIM2, IL‐1β, IL‐18, and caspase‐1 was increased in cluster 1 samples implying higher sensitivity of those samples to pyroptosis than in cluster 2 samples. Moreover, cluster 1 and cluster 2 showed significant survival outcome differences indicating the critical role of pyroptosis in GBM.

Furthermore, central nervous system tumour cell lines were labelled as cluster 1 or cluster 2 based on machine learning algorithms. GBM cell line T98G, classified as cluster 1 cells, was more sensitive to nutlin‐3 than LN229 (cluster 2 GBM cell line) as predicted. Moreover, we validated their sensitivity to pyroptosis with a potential compound, nutlin‐3, in T98G and LN229. As expected, T98G and LN229 responses to nutlin‐3 are different. Moreover, previous works reported that a novel oxidative stress marker, DHCR24, can affect cells oxidative stress to trigger pyroptosis.^51^, ^52^, ^59^ In this work, increased DHCR24 expression was found in nutlin‐3 treated cell lines implying nutlin‐3 may increase DHCR24 expression to trigger pyroptosis. In the meantime, we also found that LN229 may upregulate pyroptosis‐resistant genes and downregulate pyroptosis‐sensitive genes to respond nutlin‐3. Therefore, this is the first work that reported that nutlin3 may modulate pyroptosis through DHCR24.

As previous work reported interferon‐gamma module cells pyroptosis.^23^, ^24^, ^25^ In this work, we found that cluster 1 samples may also respond to interferon‐gamma more actively than cluster 2 which may also explain the different sensitivity of cluster 1 and 2 to pyroptosis. Meanwhile, dysregulated immune escape‐related genes expression and different tumour immunogenicity scores were also noticed between cluster 1 and cluster 2 samples. For instance, CD40, CD47, CD276, CTLA4, and PD‐L1 were upregulated in cluster 1 samples indicating their difference to immunotherapy. Moreover, a higher score on T cells immunity, glycolysis, recognition tumour, interferon‐gamma response, tumour cells recognition, and the infiltration of inhibitory immunocytes (myeloid‐derived suppressor cells and regulatory T cells), and immune checkpoint genes expression were found in cluster 1 samples than cluster 2 samples. As previously reported, the application of immunogram can be used as guidance for patients' immunotherapy.^30^, ^31^, ^47^ Meanwhile, immunogram also suggested that cluster 1 and 2 GBM samples respond to interferon‐gamma differentially. Together, GBM sensitivity to pyroptosis is associated with tumour immunosuppressive microenvironment.

The connection between GBM cells and immunocytes has been proven can modulate tumour microenvironment^65^ and response to tumour treatments.^66^, ^67^ Potential interaction between GBM cells and immunocytes based on the cluster model was also analysed. Activated macrophage/microglia normally have high expressed GRN; in the meantime, depriving GRN expression can aggravate microglia infiltration^68^ and induce macrophage to secret inflammatory associated cytokine.^69^ Moreover, inflammatory mediator IL‐6 abolished the sensitivity of GBM immunotherapy.^70^ Therefore, GRN‐SORT may affect GBM sensitivity to immunotherapy. The Cerebral Ischemic/Reperfusion Recovery model proved that EPO could promote M2 microglia differentiation and inhibit M1 microglia differentiation,^67^ implying EPO‐EPOR may contribute to constructing GBM immunosuppressive microenvironment. Chlorogenic acid has been proven that can inhibit microglia activation in LPS‐stimulated microglia,^71^ and M1 or M2 microglia were considered pro‐ or anti‐inflammatory phenotypes.^72^ Taken together, cluster 2 and cluster 1 neoplastic may modulate microglia polarization to affect GBM immunogenicity and tumour sensitivity to immunotherapy.

However, no sufficient studies to support the potential relationship between microglia and GBM cells through ligand‐receptor pairs like GDF2‐(ACVRL1 + BMPR2), TNFSF12‐TNFRSF12A, NAMPT‐(ITGA5 + ITGB1). Those pairs may be novel pathways that were able to regulate microglia activation, differentiation, or polarization and through which to affect GBM prognosis. For instance, NAMPT can be secreted from microglia,^73^ and its inhibitor can prolong GBM‐bearing animals' survival time on accompany by PD‐1 checkpoint blockade.^74^ TNFSF12‐TNFRSF12A can modulate glioma progression^75^, ^76^ and may participate in microglia activation.^77^ GDF2‐ACVRL1 can modulate glioma angiogenesis,^78^ but its association with microglia was elusive. Besides, EGFR,^79^ SPP1‐CD44,^80^ OSM^81^ were also reported can affect microglia phenotype in glioma. Moreover, previous studies reported that pyroptosis can affect microglia activation.^82^, ^83^ Therefore, GBM sensitivity to pyroptosis may affect microglia differentiation and activation but its potential mechanism is elusive.

In summary, this work proposed the subtype of GBM based on pyroptosis‐associated regulators. Cluster 1 phenotype was recognized as a malignant subtype of GBM (mesenchymal and classical) along with a poor prognosis. Interestingly, the cluster 2 phenotype seems to interact with microglia through multiple pathways and participate in microglia polarization, while cluster 1 phenotype manifested higher immunogenicity and immune escape‐related genes expression. In vitro validation further confirmed that cluster 1 samples are more sensitive to nutlin‐3, and targeting pyroptosis may be a novel option for treating GBM, especially for the malignant subtype. Considering the development of novel methods like patients‐derived GBM organoids and xenograft, further exploring the effect of nutlin3 in GBM with those methods may help better understanding.^84^

## AUTHOR CONTRIBUTIONS

**Zeyu Wang:** Writing – original draft; writing – review and editing; data curation; formal analysis; visualization; methodology. **Ziyu Dai:** Data curation; formal analysis; validation. **Hao Zhang:** Validation. **Nan Zhang:** Validation. **Xisong Liang:** Validation. **Luo Peng:** Methodology. **Jian Zhang:** Visualization. **Zaoqu Liu:** Visualization. **Yun Peng:** Methodology. **Quan Cheng:** Funding acquisition; project administration; supervision. **Zhixiong Liu:** Funding acquisition; supervision.

## ACKNOWLEDGEMENTS

This work was supported by the National Nature Science Foundation of China (82073893, 82172685, 81873635, 81703622); Hunan Provincial Natural Science Foundation of China (2022JJ70078, 2022JJ20095), Hunan Provincial Health Committee Foundation of China (202204044869) and Xiangya Hospital Central South University postdoctoral foundation. We are grateful to the High Performance Computing Center of Central South University for partial support of this work.

## CONFLICT OF INTEREST

All authors have no conflicts of interest to be declared.

## Supporting information


**Figure S1.** The construction of the cluster model in the Xiangya cohort, the TCGA GBM array data, and the GBM metadata. (A) The principal component analysis of the cluster model. (B) The consensus clustering matrix of the cluster model. (C) The cumulative distribution function curve of the cluster model.

**Figure S2.** The expression profile of pyroptosis‐related genes in the Xiangya cohort, including AIM2, APIP, CASP1, CASP3, CASP4, CASP8, CASP9, DDX58, IFI16, IL1B, IL18, MAPK8, MAPK9, NAIP, NFKB1, NFKB2, NLRP1, and NLRP3.

**Figure S3.** Survival analysis of the cluster model and its association with GBM clinical features. (A) Disease‐specific survival analysis in TCGA GBM array data. (B) Progression free interval survival analysis in TCGA GBM array data. Association of the cluster model with GBM clinical features in TCGA GBM array data (C) and GBM metadata (D).

**Figure S4.** Biofunction prediction in the TCGA GBM array data and GBM metadata. (A) GO and KEGG enrichment analysis based on the GSEA analysis in the TCGA GBM array data. (B) GO and KEGG enrichment analysis based on the GSEA analysis in the GBM metadata.

**Figure S5.** Immunogram in the TCGA GBM array data, GBM metadata, and single‐cell RNAseq data. (A) The construction of immunogram (version 2017 and version 2020) in the GBM metadata. (B) The construction of immunogram (version 2017 and version 2020) in the TCGA GBM array data. (C) The immunogram in the single cell RNA‐seq data.

**Figure S6.** Immune checkpoint genes expression profile in the single cell RNA‐seq data, TCGA GBM array data, and GBM metadata. (A) Immune checkpoint genes expression profile in the single cell RNAseq data. (B) Immune checkpoint genes expression profile in the TCGA GBM array data. (C) Immune checkpoint genes expression profile in the GBM metadata.

**Figure S7.** Immune landscape difference in the cluster model in the TCGA GBM array data. (A) Correlation of tumour purity with a stromal score or immune score was calculated by performing the ESTIMATE algorithm. (B) Immunocytes infiltration was analysed by using the CIBERSORT algorithm and xCell analysis.

**Figure S8.** Immune landscape difference in the cluster model in the GBM metadata. (A) Correlation of tumour purity with a stromal score or immune score was calculated by performing the ESTIMATE algorithm. (B) Immunocytes infiltration was analysed by using CIBERSORT algorithm and xCell analysis.

**Figure S9.** Cell–cell communication difference in the cluster model. (A) HBEGF‐EGFR in the EGF signalling pathway. (B) SPP1‐CD44 in the SPP1 signalling pathway. (C) OSM‐(LIFR+IL6ST) in the OSM signalling pathway. (D) FASL–FAS in the FASLG signalling pathway. (E) IGF1‐(ITGA6 + ITGB4) in the IGF signalling pathway. (F) IL6‐(IL6R + IL6ST) in the IL6 signalling pathway.

**Figure S10.** Cell–cell communication difference in the cluster model. (A) NGF‐NGFR in the NGF signalling pathway. (B) KITL‐KIT in the KIT signalling pathway. (C) KLK3‐NGFR in the NGF signalling pathway. (D) GZMA‐F2R in the PARs signalling pathway. (E) The expression of pryoptosis‐related genes that are upregulated in LN229 but not in T98G. (F) The expression of pryoptosis‐related genes that are upregulated in T98G but not in LN229. (G) The expression of pryoptosis‐related genes that are downregulated in LN229 but not in T98G. NS, no significant; **p < 0.01; ***p < 0.001.

Table S1.

## Data Availability

The dataset(s) supporting the conclusions of this article are available in the TCGA, CGGA and GEO databases. The xiangya GBM cohort (ID: HRA001618) can be downloaded from China National Center for Bioinformation (https://www.cncb.ac.cn/). Other datasets generated in the current study could be available by contacting the corresponding author.
