# Supplementary figures and images for: Comprehensive analysis of pyroptosis‐related gene signatures for glioblastoma immune microenvironment and target therapy
Source: Cell Prolif. 2023 Jan 21;56(3):e13376. doi: 10.1111/cpr.13376 (PMC9977674; doi:10.1111/cpr.13376)

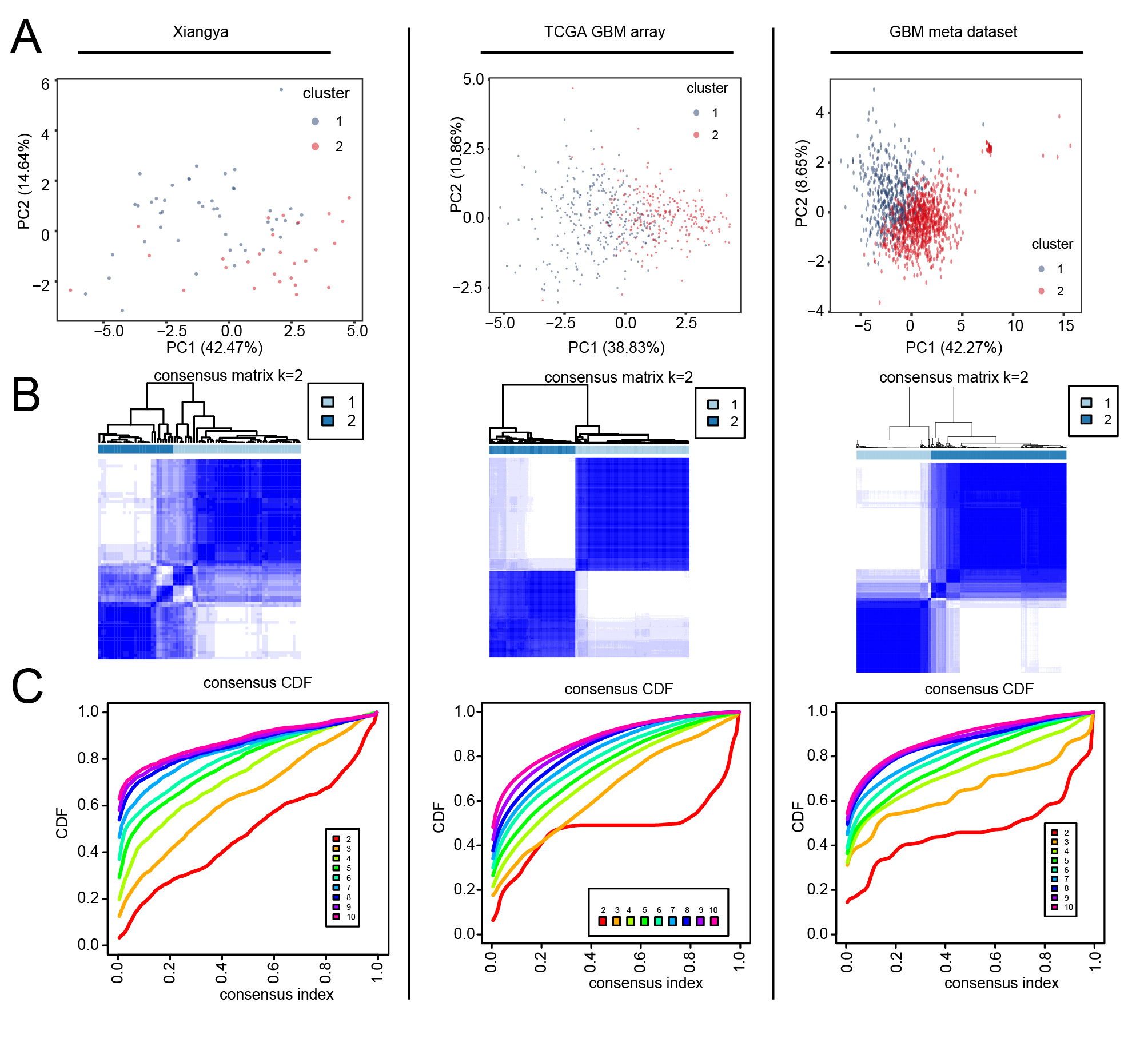

Supplement: Supplementary file 1 — Figure S1. The construction of the cluster model in the Xiangya cohort, the TCGA GBM array data, and the GBM metadata. (A) The principal component analysis of the cluster model. (B) The consensus clustering matrix of the cluster model. (C) The cumulative distribution function curve of the cluster model. [file CPR-56-e13376-s005.jpg]

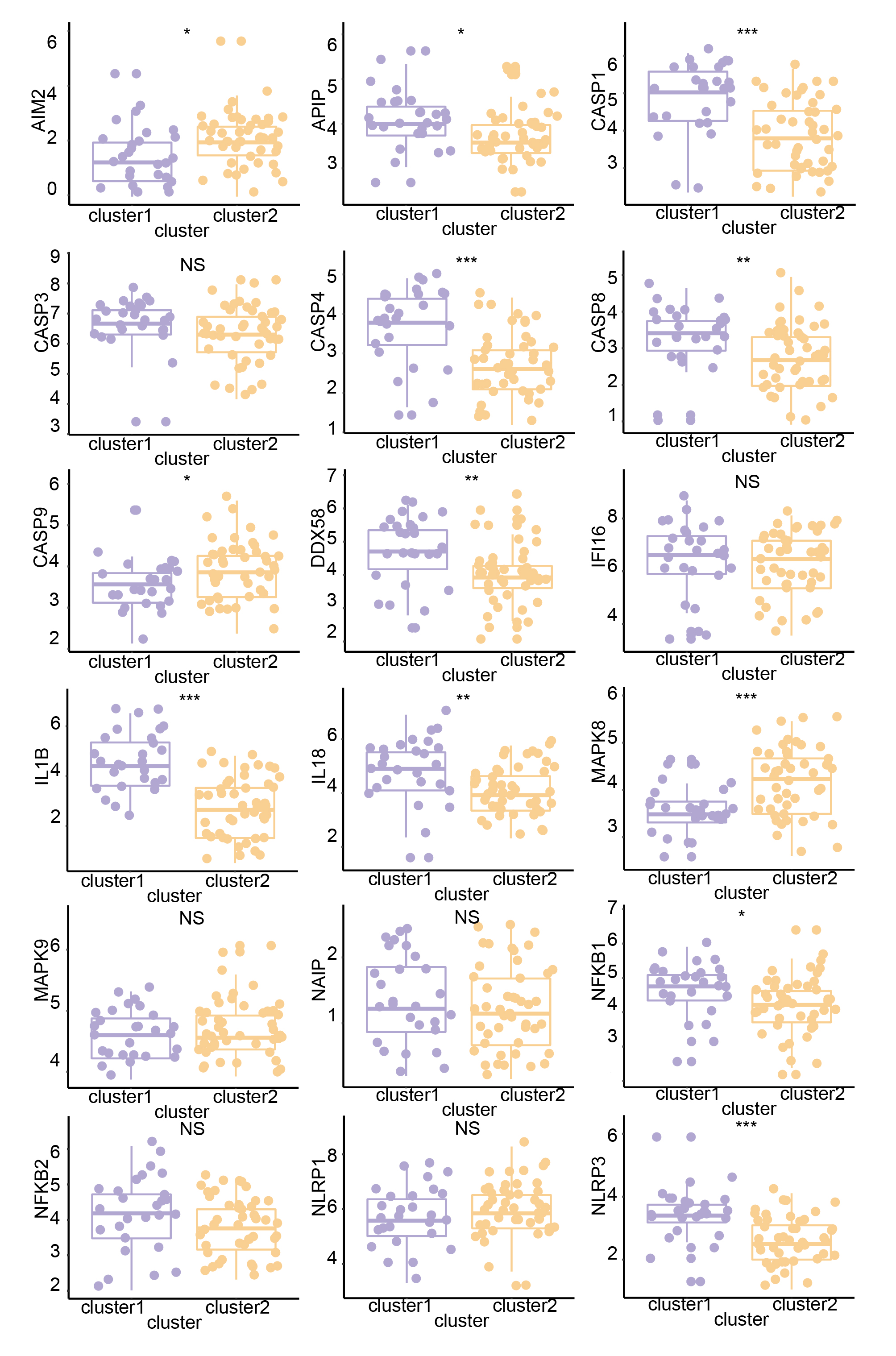

Supplement: Supplementary file 2 — Figure S2. The expression profile of pyroptosis‐related genes in the Xiangya cohort, including AIM2, APIP, CASP1, CASP3, CASP4, CASP8, CASP9, DDX58, IFI16, IL1B, IL18, MAPK8, MAPK9, NAIP, NFKB1, NFKB2, NLRP1, and NLRP3. [file CPR-56-e13376-s004.jpg]

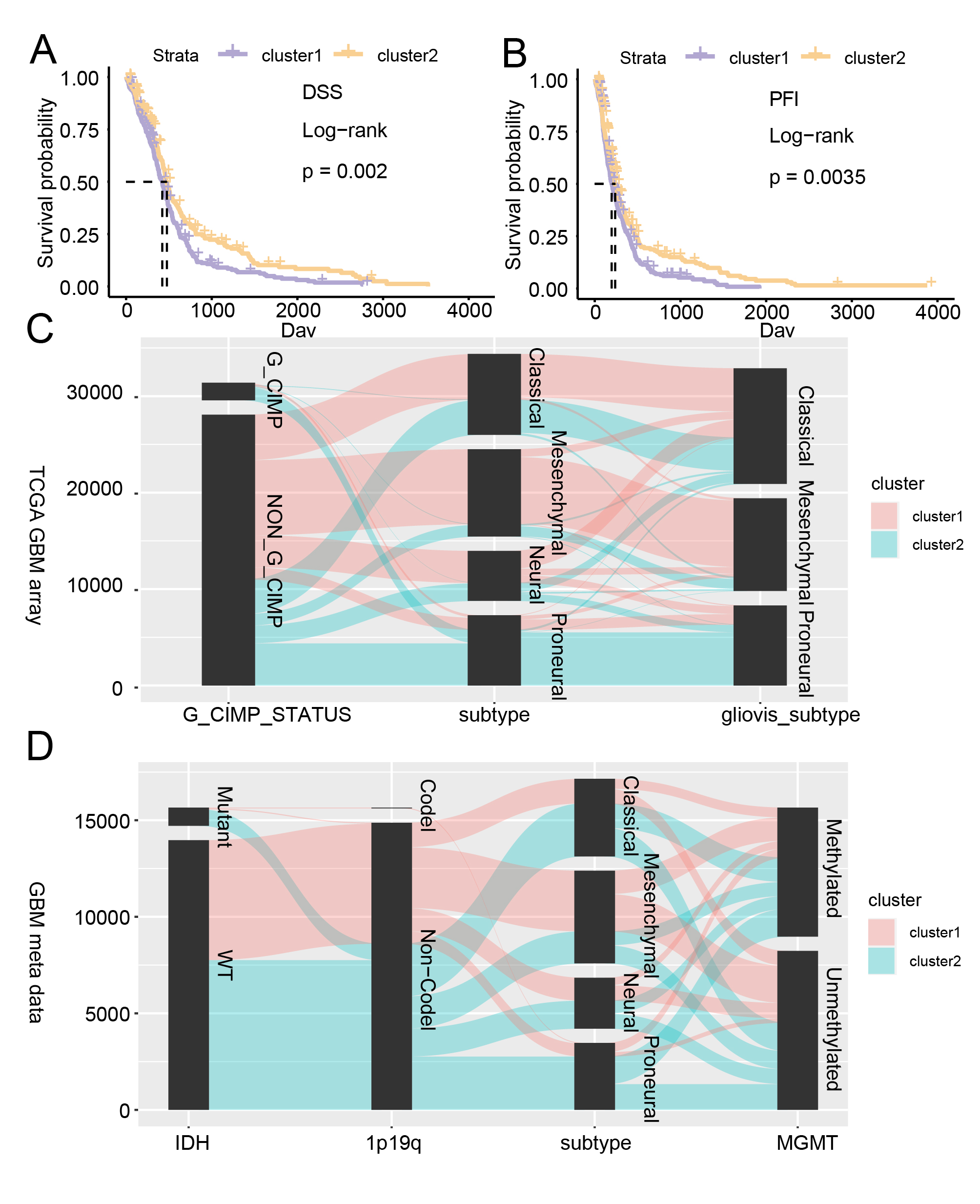

Supplement: Supplementary file 3 — Figure S3. Survival analysis of the cluster model and its association with GBM clinical features. (A) Disease‐specific survival analysis in TCGA GBM array data. (B) Progression free interval survival analysis in TCGA GBM array data. Association of the cluster model with GBM clinical features in TCGA GBM array data (C) and GBM metadata (D). [file CPR-56-e13376-s011.jpg]

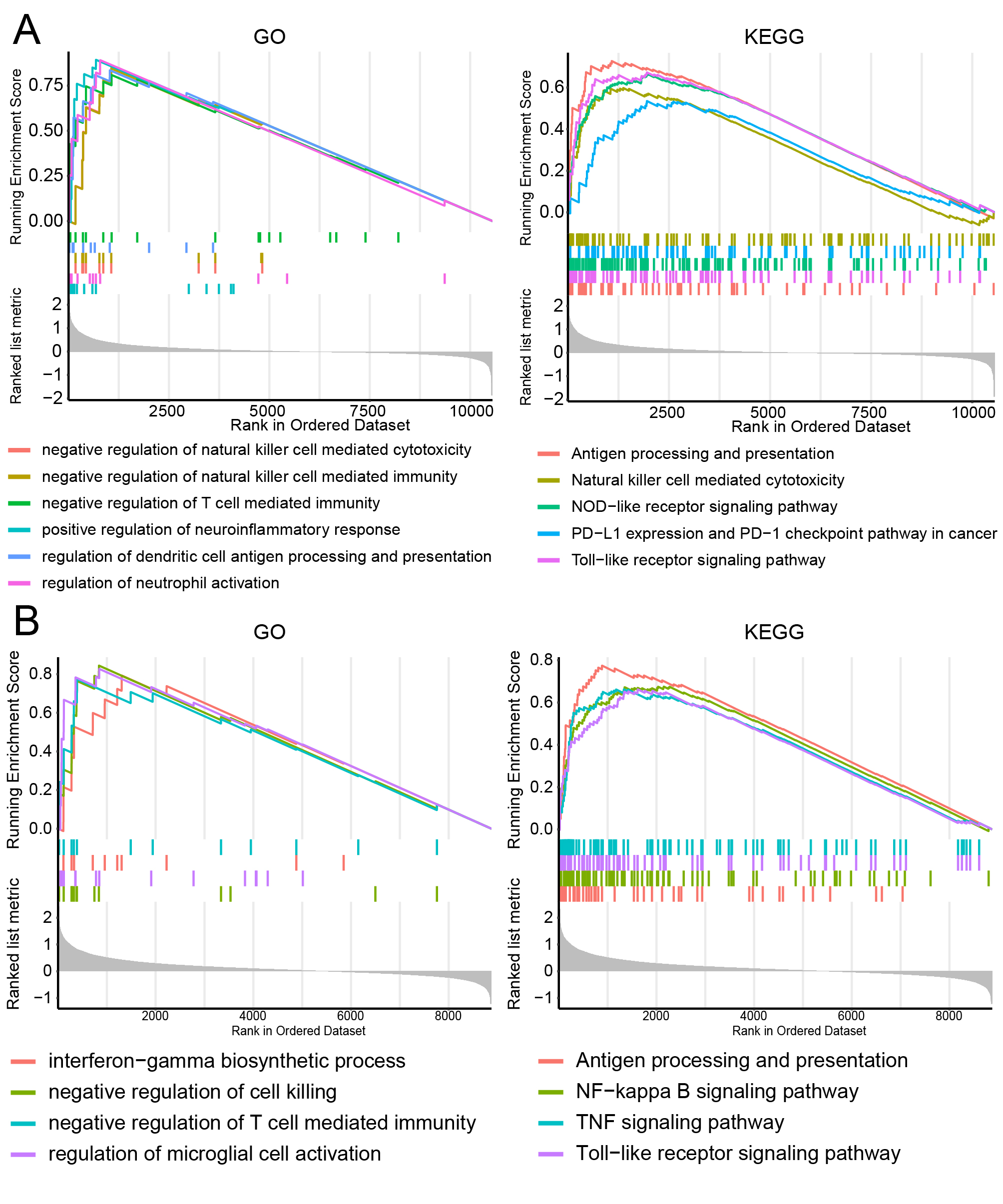

Supplement: Supplementary file 4 — Figure S4. Biofunction prediction in the TCGA GBM array data and GBM metadata. (A) GO and KEGG enrichment analysis based on the GSEA analysis in the TCGA GBM array data. (B) GO and KEGG enrichment analysis based on the GSEA analysis in the GBM metadata. [file CPR-56-e13376-s002.jpg]

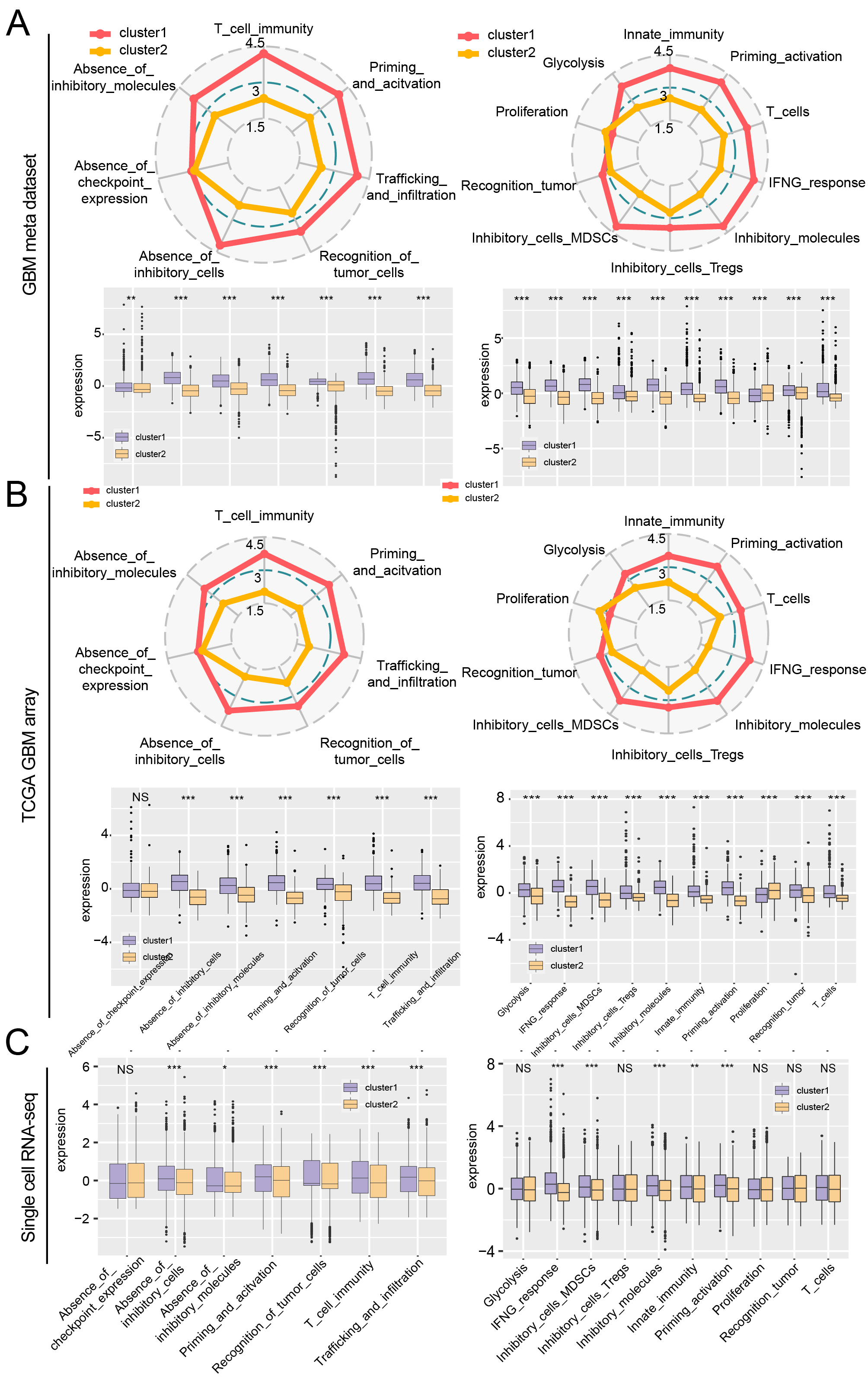

Supplement: Supplementary file 5 — Figure S5. Immunogram in the TCGA GBM array data, GBM metadata, and single‐cell RNAseq data. (A) The construction of immunogram (version 2017 and version 2020) in the GBM metadata. (B) The construction of immunogram (version 2017 and version 2020) in the TCGA GBM array data. (C) The immunogram in the single cell RNA‐seq data. [file CPR-56-e13376-s007.jpg]

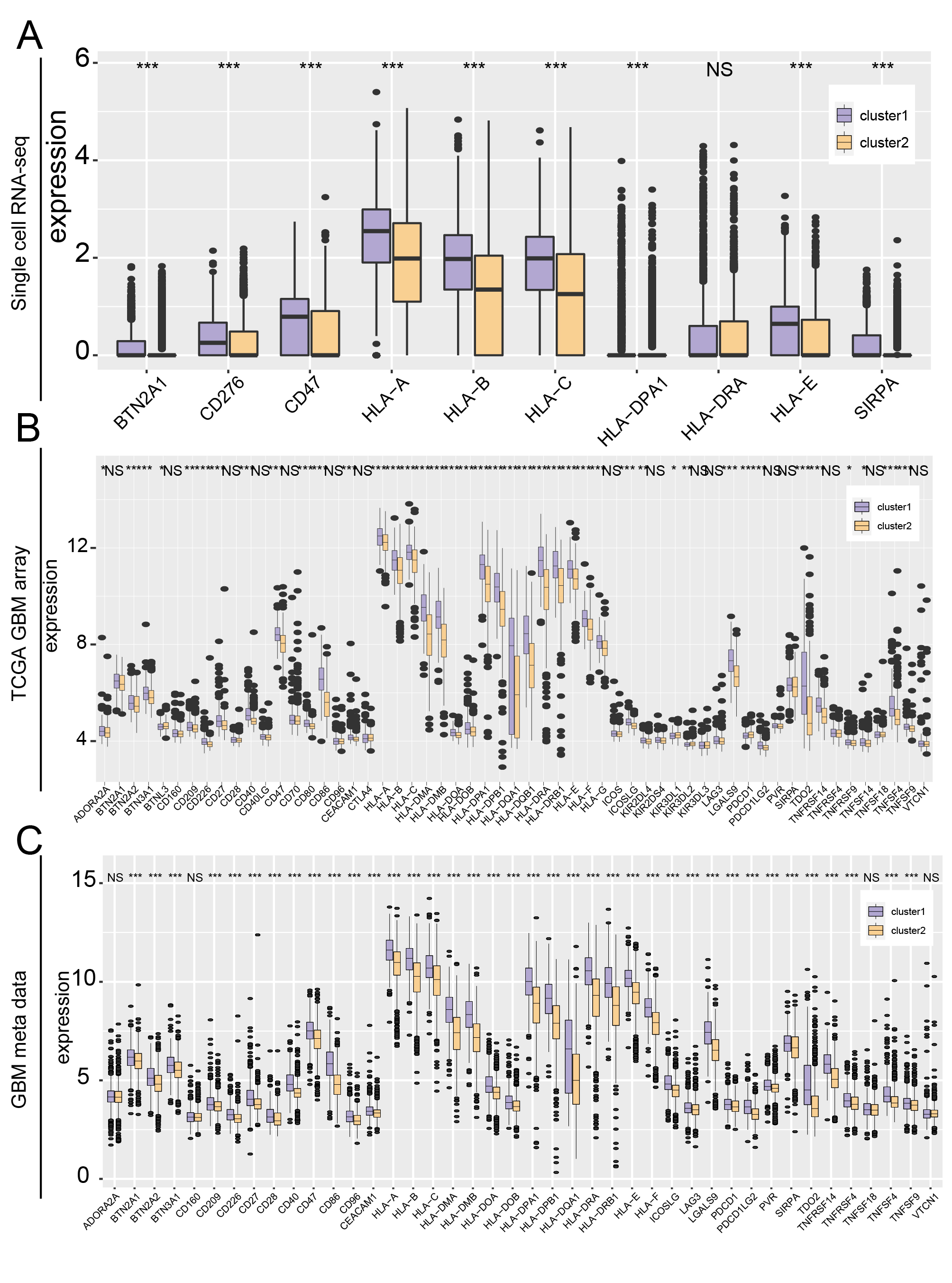

Supplement: Supplementary file 6 — Figure S6. Immune checkpoint genes expression profile in the single cell RNA‐seq data, TCGA GBM array data, and GBM metadata. (A) Immune checkpoint genes expression profile in the single cell RNAseq data. (B) Immune checkpoint genes expression profile in the TCGA GBM array data. (C) Immune checkpoint genes expression profile in the GBM metadata. [file CPR-56-e13376-s008.jpg]

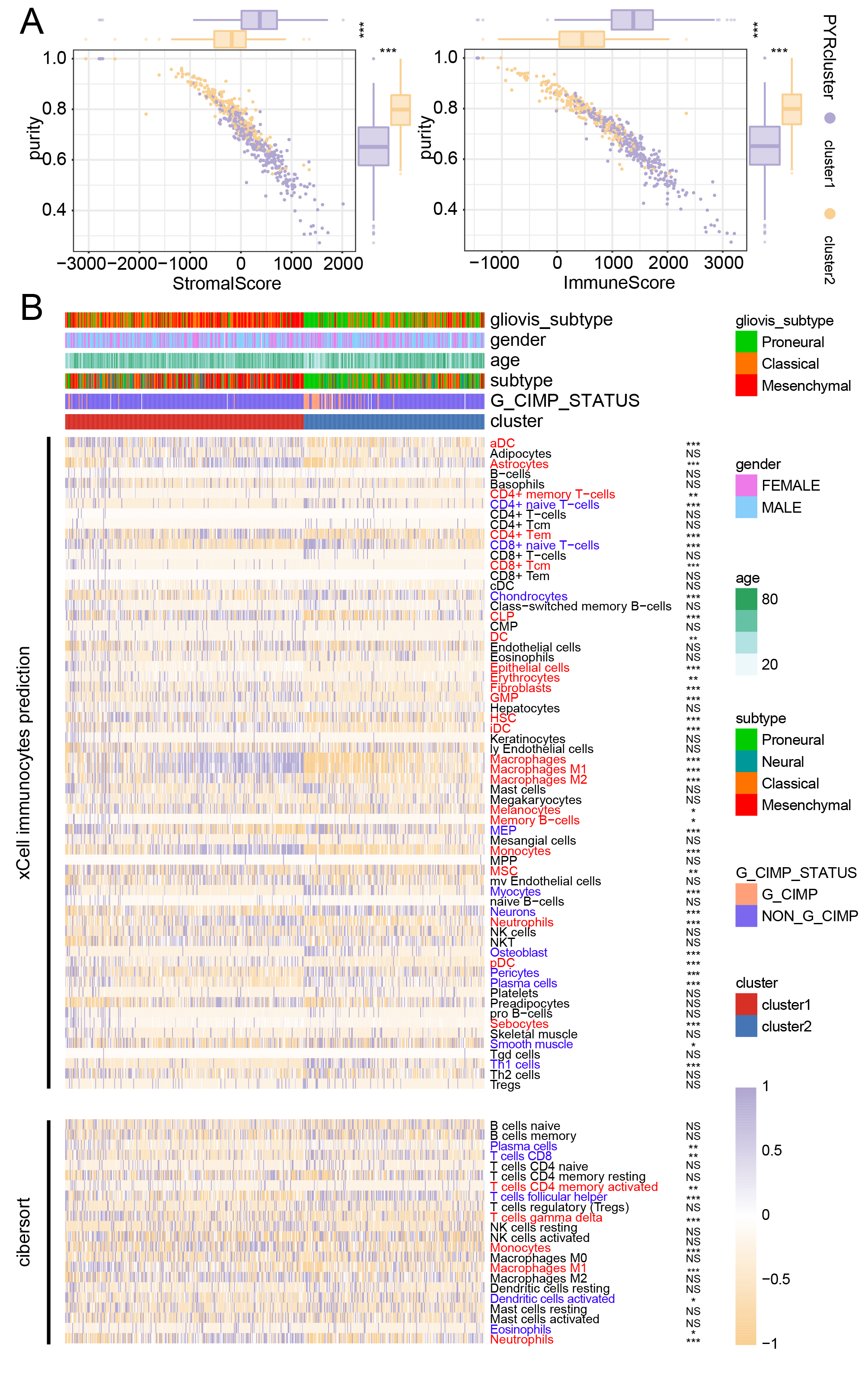

Supplement: Supplementary file 7 — Figure S7. Immune landscape difference in the cluster model in the TCGA GBM array data. (A) Correlation of tumour purity with a stromal score or immune score was calculated by performing the ESTIMATE algorithm. (B) Immunocytes infiltration was analysed by using the CIBERSORT algorithm and xCell analysis. [file CPR-56-e13376-s001.jpg]

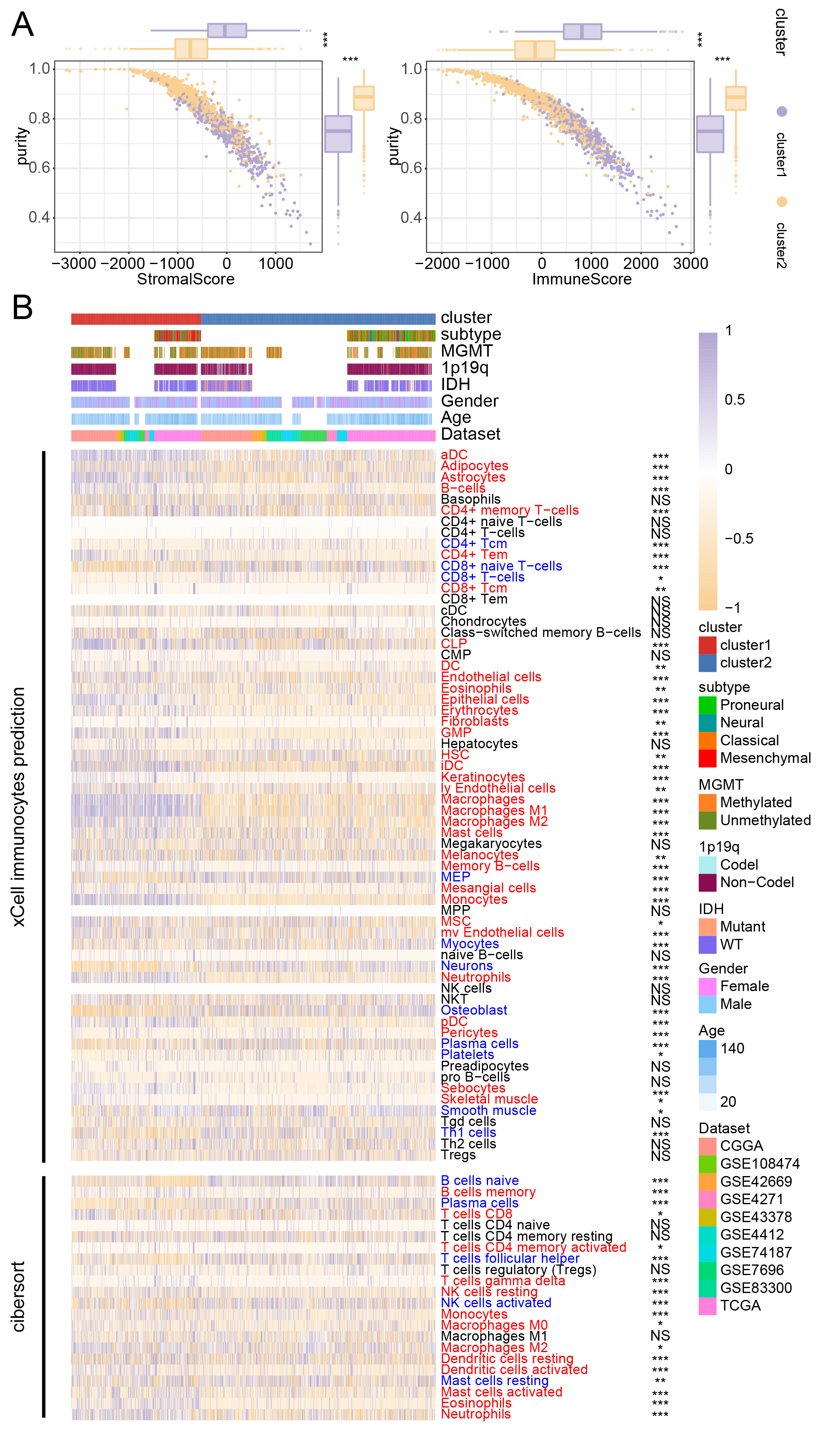

Supplement: Supplementary file 8 — Figure S8. Immune landscape difference in the cluster model in the GBM metadata. (A) Correlation of tumour purity with a stromal score or immune score was calculated by performing the ESTIMATE algorithm. (B) Immunocytes infiltration was analysed by using CIBERSORT algorithm and xCell analysis. [file CPR-56-e13376-s009.jpg]

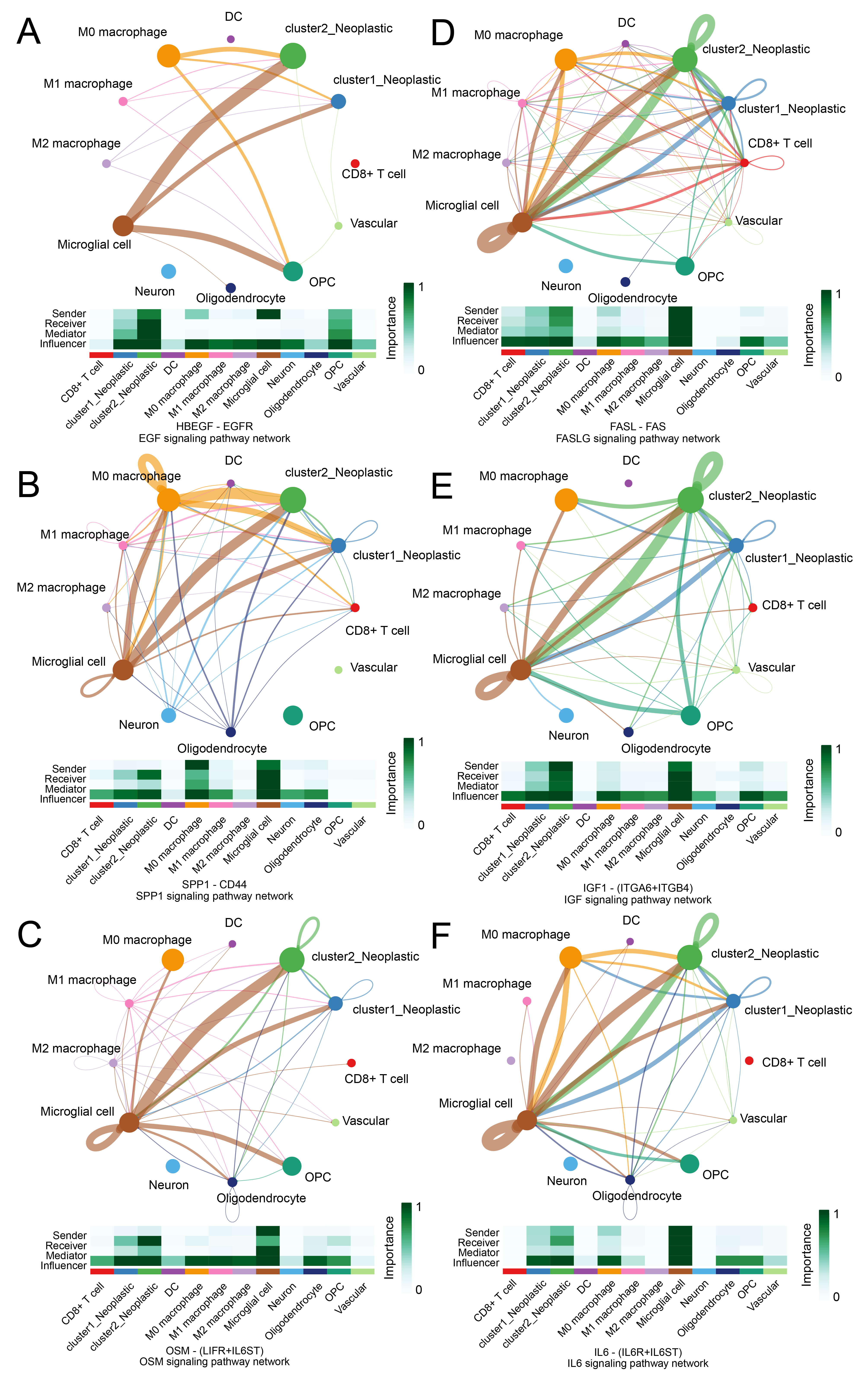

Supplement: Supplementary file 9 — Figure S9. Cell–cell communication difference in the cluster model. (A) HBEGF‐EGFR in the EGF signalling pathway. (B) SPP1‐CD44 in the SPP1 signalling pathway. (C) OSM‐(LIFR+IL6ST) in the OSM signalling pathway. (D) FASL–FAS in the FASLG signalling pathway. (E) IGF1‐(ITGA6 + ITGB4) in the IGF signalling pathway. (F) IL6‐(IL6R + IL6ST) in the IL6 signalling pathway. [file CPR-56-e13376-s006.jpg]

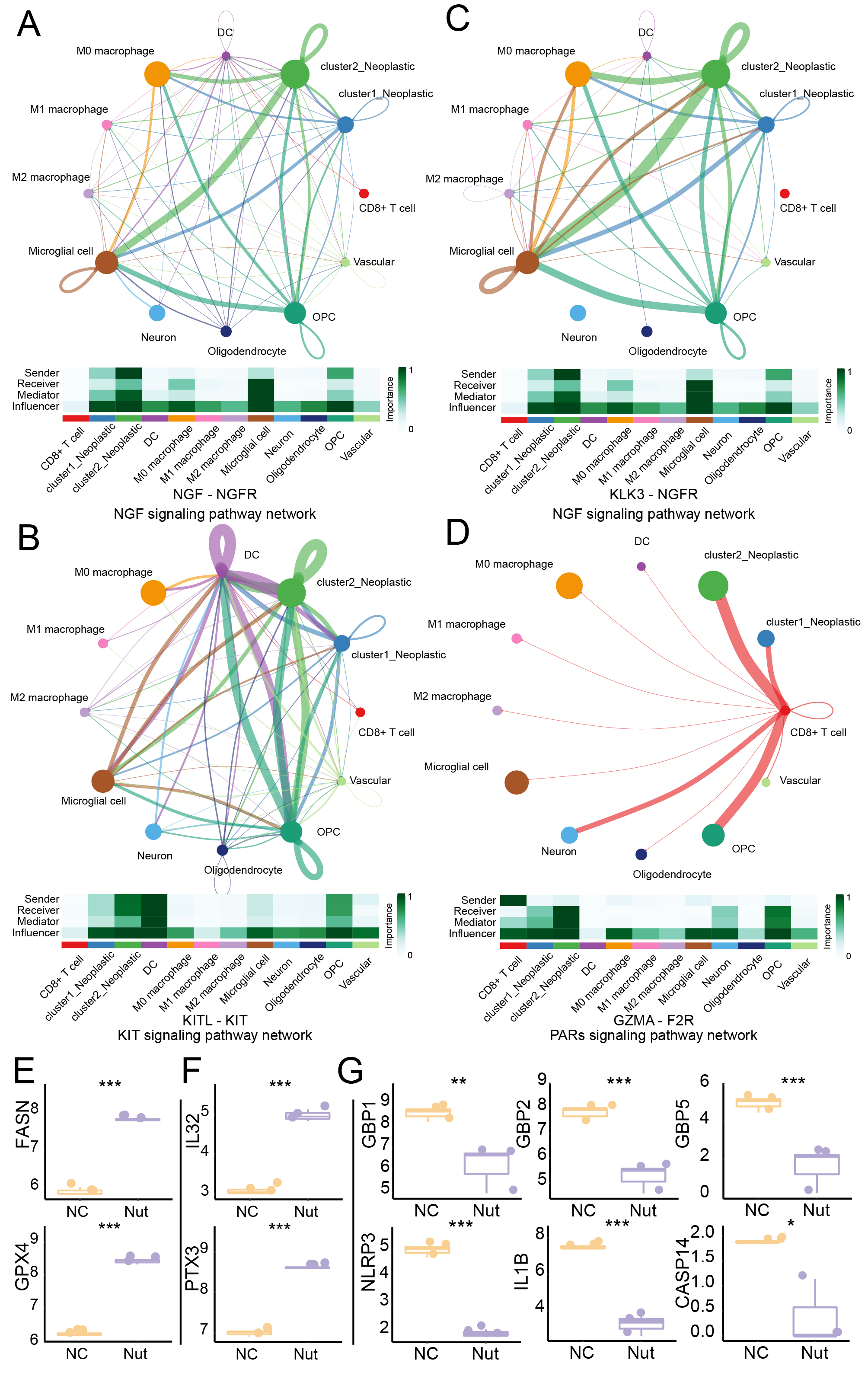

Supplement: Supplementary file 10 — Figure S10. Cell–cell communication difference in the cluster model. (A) NGF‐NGFR in the NGF signalling pathway. (B) KITL‐KIT in the KIT signalling pathway. (C) KLK3‐NGFR in the NGF signalling pathway. (D) GZMA‐F2R in the PARs signalling pathway. (E) The expression of pryoptosis‐related genes that are upregulated in LN229 but not in T98G. (F) The expression of pryoptosis‐related genes that are upregulated in T98G but not in LN229. (G) The expression of pryoptosis‐related genes that are downregulated in LN229 but not in T98G. NS, no significant; **p < 0.01; ***p < 0.001. [file CPR-56-e13376-s003.jpg]
